# A CSE-containing promoter region of *AcoINV3* is associated with 15℃-induced pineapple translucency during fruit enlargement

**DOI:** 10.1186/s12870-026-09737-7

**Published:** 2026-08-12

**Authors:** Haiyan Shu, Aiping Luan, Wei Sun, You Wang, Junhu He, Rulin Zhan, Shenghe Chang

**Affiliations:** 1 Tropical Crops Genetic Resources Institute, Chinese Academy of Tropical Agricultural Sciences, Haikou, 571101 China; 2 Sanya Research Institute, Chinese Academy of Tropical Agricultural Sciences, Sanya, 572025 China; 3 State Key Laboratory of Tropical Crop Breeding, Sanya, 572025 China; 4 Key Laboratory of Crop Gene Resources and Germplasm Enhancement in Southern China, Ministry of Agriculture and Rual Affairs, Haikou, 571101 China; 5 Key Laboratory of Tropical Crops Germplasm Resources Genetic Improvement and Innovation of Hainan Province, Haikou, 571101 China

**Keywords:** Pineapple, Chilling, Translucency, Fruit weight, Cell wall invertase, Cold shock element

## Abstract

**Background:**

Pineapple is the third most widely cultivated tropical fruit globally and the fourth in China. Annual losses due to translucency account for approximately 20% of China’s pineapple production. Studying its mechanism and developing controlling strategies is important for the industry. Chilling during fruit enlargement often leads to severe translucency, and larger fruits tend to show higher incidence of translucency. However, the underlying mechanisms remain unclear.

**Results:**

15℃ treatment during fruit enlargement increased apoplastic fructose and glucose levels and up-regulated expression of the cell wall invertase gene *AcoINV3*. The fructose and glucose content in pulp intercellular space of *AcoINV3* mutant MU-2 is significantly less than that of the pineapple transformed with the empty vector. These values of MU-2 treated with 15 ℃ were similar with those of MU-2 always grown in 25℃ greenhouse. Under 15℃-stress, MU-2 exhibited significantly lower translucency rates than empty-vector control. Its single-fruit weight was also much less than the control. A cold shock element (CSE, 5′-CCGAC-3′) in the *AcoINV3* promoter was identified as a 15℃-responsive *cis*-element. The *AcoINV3* CSE-knockout line CSE-3 exhibited a significantly lower incidence of translucency than wild-type plants when subjected to 15℃ during fruit enlargement. Its single-fruit weight was comparable to that of wild-type plants under 25℃ conditions.

**Conclusions:**

These results suggest that 15℃ treatment during fruit enlargement induces translucency and promotes fruit weight. This inducement was associated with the CSE in the *AcoINV3* promoter. The CSE in the *AcoINV3* promoter has 15℃-responsive function. Targeted knockout of the CSE reduces translucency incidence without compromising single-fruit weight under 15℃ condition.

**Supplementary Information:**

The online version contains supplementary material available at https://doi.org/10.1186/s12870-026-09737-7.

## Introduction

Flesh translucency is a major physiological disorder that severely compromises the commercial value of pineapples (*Ananas comosus*), rendering affected fruits unmarketable due to a water-soaked appearance, loose texture, and liquid-filled intercellular spaces [1, 2]. This disorder is closely linked to pulp calcium content, sugar accumulation, and chilling exposure during the fruit enlargement phase [3–9]. Agronomic interventions, such as increasing pulp calcium levels, pre-harvest leaf pruning, and retention of the crown at harvest, have been shown to mitigate its incidence [2, 10]. Recent studies also suggest potential involvement of CCCH zinc finger genes in pineapple translucency [11].

Pineapple translucency predominantly initiates 2–3 months prior to harvest, coinciding with the rapid volumetric expansion of the fruit [2]. The translucency incidence of the ‘Smooth Cayenne’ cultivar doubled from 20% to 40% when minimum temperatures dropped from 20 °C to 15 °C during the pre-harvest trimester [5]. The correlation between translucency incidence rates and temperature during fruit enlargement stage is also evident in China’s main production regions. Meteorological data from Zhanjiang (the largest pineapple producing region in China) (Guangdong Weather Report) reveal that minimum temperatures during the fruit enlargement stage (February) were 18 °C (2019), 14 °C (2020), and 10 °C (2021). Correspondingly, local translucency incidence rates escalated from 15% to 24% and 44% over these three years [12], underscoring the pivotal role of chilling stress in triggering widespread outbreaks.

Adjusting the planting schedule to circumvent chilling stress during fruit enlargement appeared to be a viable strategy; however, it inevitably synchronizes the cropping cycle with regional peak rainfall periods. In Guangdong (May-September) and Hainan (May-October) (China Meteorological Data Network), such temporal overlap significantly elevates the risk of large-scale outbreaks of heart rot and pineapple wilt disease [13]. Elucidating the mechanisms underlying flesh translucency and breeding resistant cultivars remain fundamental strategies for sustainably resolving this issue.

Field observations consistently indicated a positive correlation between fruit size and translucency incidence [1, 9]. To mitigate this disorder, growers often resort to sacrificing yield for quality by removing partial leaves to suppress fruit expansion [9]. This trade-off implies an intrinsic link between translucency development, carbohydrate metabolism, and the fruit expansion process. The induction of translucency by chilling might be mediated by sugar accumulation in fruit. The activities of cell-wall invertase, electrolyte leakage, sucrose content in flesh increased fast during 0 to 4 weeks before harvest [9]. It has been proposed that enhanced cell-wall invertase activity promotes sucrose unloading into the apoplast, increasing solute concentration and driving water influx, thereby reducing porosity and inducing translucency [9]. However, these remain speculative. The absence of comparative physiological data between normal and translucent fruits, coupled with a lack of direct investigation into the causal relationship between cell-wall invertase genes and translucency, limits the validity of these descriptions [9].

Sucrose, glucose, and fructose were the main sugars in pineapple fruit [9]. They all exhibit hygroscopic properties, which can induce water soaking in fleshy fruits [14]. Fructose and glucose possess significantly higher water-holding capacities than sucrose; at 22 °C, their respective capacities are approximately 90 mL/mol, 54 mL/mol, and 13.68 mL/mol [14–17]. The fructose and glucose contents in the intercellular spaces in translucent fruit pulp were significantly higher than those in normal fruit, whereas the levels in the whole pulp showed no significant difference [3]. Decreasing hexose content in intercellular space of pineapple fruit may mitigate translucency incidence. The accumulation of fructose and glucose in fruit results from sucrose hydrolysis, a reaction catalyzed by invertase [18, 19]. Plants harbor two primary classes of invertases: acid invertases (comprising cell wall and vacuolar isoforms) and neutral/alkaline (cytoplasmic) invertases [20, 21]. Only cell wall invertase mediates sucrose hydrolysis within the apoplast, releasing glucose and fructose into the intercellular spaces [22]. Mutating cell wall invertase genes, holds promise for reducing apoplastic solute concentration and effectively lowering the incidence of pineapple translucency compared to wild-type controls.

This study investigated the expression dynamics of a cell wall invertase gene, *AcoINV3*, in pineapple pulp under 15℃ stress during fruit enlargement. Promoter analysis revealed a cold-shock element (CSE) within the *AcoINV3* regulatory region. Subsequently, *AcoINV3* mutants and CSE-knockout lines were generated via genome editing. By characterizing the interplay between 15℃ treatment, *AcoINV3* expression, fruit weight, and translucency incidence, this work establishes a theoretical and genetic foundation for breeding pineapple cultivars that combine translucency resistance with high yield.

## Materials and methods

### Plant material

The pineapple cultivar used in this study was Tainung No. 17 (TN17), also known as ‘Jinzuan’ (Golden Diamond). This hybrid cultivar was developed by the Chiayi Agricultural Experiment Station in Taiwan, China. TN17 exhibits a semi-erect growth habit with a plant height of approximately 70 cm and relatively vertical leaf growth. Its leaf margins are spineless or bear few spines, facilitating field management. The fruit is characterized by high total soluble solids (TSS), superior taste, and excellent overall quality. The recommended planting density is 42,000–45,000 plants per hectare, with an average yield of 30-37.5 tons per hectare. TN17 is one of the new pineapple cultivars actively promoted by the Hainan Provincial Department of Agriculture and Rural Affairs, China. The pineapples used in this study were planted at the Experimental Field of Haikou Experimental Station, Chinese Academy of Tropical Agricultural Sciences, Danzhou, China.

Plants were subjected to temperature treatments in intelligent glass greenhouses (Qingzhou Rongguang Greenhouse Engineering Co., Ltd., China). These facilities employ a PLC/PID-based closed-loop climate control system integrating active heating/cooling sources and high-efficiency thermal insulation. Air temperatures were maintained at either 15 ± 1 °C or 25 ± 1 °C. To mitigate intense solar radiation and high ambient heat, cooling at 25 °C was achieved via mechanical forced ventilation combined with a water-cooled chiller unit supplying 7–12 °C chilled water to fan coil units. At 15 °C, the system utilized the reversible chiller unit in heat pump mode for heating, switching to mechanical ventilation and active cooling when necessary to prevent overheating. Critically, no artificial lighting or light-deprivation shading was applied; thus, plants were exposed to the natural photoperiod of Hainan Province (11–13 h light), ensuring that observed physiological responses was driven by temperature rather than photoperiod manipulation.

### Sugar content determination

Apoplastic fluid (AF) was extracted following the vacuum infiltration-centrifugation method described by Versluys et al. (2022) [23] with minor modifications. Fresh pineapple pulp was cut into 1-cm cubes and washed with distilled water to remove damaged tissues and surface sugars. The samples were incubated in ice-cold infiltration buffer (20 mM NaOAc, pH 5.0; 400 µM rhamnose as internal standard; 100 µM PMSF) and subjected to vacuum infiltration twice at 45 mbar for 2 min each, with slow release of pressure between cycles. The infiltrated pulp was rinsed with ddH₂O, gently blotted dry with paper towels, wrapped in Parafilm, and placed in a 5-mL syringe inside a 14-mL tube. After centrifugation at 950 × g for 10 min at 4 °C, the collected AF was transferred to clean 1.5-mL Eppendorf tubes and kept on ice. The AF was boiled at 100 °C for 10 min to inactivate enzymes and purified using a Dowex^®^ column with H⁺ and acetate resins to remove salts and organic acids.

Sucrose, glucose, and fructose concentrations were determined by high-performance liquid chromatography (HPLC) using a Hitachi L-2130 pump coupled with a Hitachi L-2490 refractive index (RI) detector. Separation was achieved on a Waters Sugar-Pak I column (6.5 × 300 mm, 10 μm, calcium-form cation exchange resin) maintained at 85 °C. The mobile phase was ultrapure water (Milli-Q, 18.2 MΩ·cm) delivered at a flow rate of 0.5 mL/min. The injection volume was 10 µL. Standard curves were prepared using HPLC-grade sucrose, glucose, and fructose (Sigma-Aldrich) at concentrations ranging from 0.1 to 20 mg/mL. All calibration curves exhibited excellent linearity (R² > 0.999). The limits of detection (LOD, S/*N* = 3) were 0.05 mg/mL for glucose, 0.04 mg/mL for fructose, and 0.03 mg/mL for sucrose. The limits of quantification (LOQ, S/*N* = 10) were 0.15, 0.12, and 0.10 mg/mL, respectively. Recovery rates, which were determined by spiking known amounts of standards into AF samples, range from 95.2% to 102.8% (RSD < 3%). Sugar concentrations in AF were corrected by the apoplast dilution factor (ADF), calculated from the rhamnose concentration change in the infiltration buffer before and after infiltration, and expressed as µg per gram fresh weight (µg/g FW) of original pulp tissue.

After pulp cubes were centrifugated at 950 × g for 10 min at 4 °C and the AF was collected, the pulp cubes were centrifugated at 5000 × g for 10 min at 4 °C and the intracellular liquid was collected. Sugar content in intracellular liquid was measured as described in AF.

### Vector construction for pineapple *AcoINV3* mutant development

Plasmids pYLsgRNA-OsU3m (Plasmid66193) and pYLCRISPR/Cas9Pubi-H (Plasmid66187) were obtained from Addgene (USA). Vector construction was performed as described previously [24] with modifications. The 20-bp target sequence was 5′-GCGCAAGATTGCGGGGACTT-3′. Complementary oligonucleotides Oligo A (5′-GATTGCGCAAGATTGCGGGGACTTGTTTTAGAGCTAGAAATAGC-3′) and Oligo B (5′-AAACAAGTCCCCGCAATCTTGCGATC-3′) were synthesized and annealed by heating at 95 °C for 5 min, followed by gradual cooling to 25 °C at 0.1 °C/min. The annealed oligonucleotide (diluted 10-fold) was ligated into BsaI-digested pYLsgRNA-OsU3m in a 20-µL reaction containing 50–100 ng plasmid, 1 µL diluted oligo, 1 µL BsaI-HFv2, 1 µL T4 DNA ligase, and 2 µL 10× T4 DNA ligase buffer. The reaction was subjected to 20 cycles of 37 °C/5 min and 16 °C/10 min, followed by 37 °C/30 min and 80 °C/10 min. The product was transformed into *E. coli* DH5α competent cells and selected on LB medium with 50 µg/mL spectinomycin. Positive clones were verified by sequencing using the OsU3 promoter universal primer, and the confirmed plasmid was designated pYLsgRNA-*AcoINV3*. The sgRNA expression cassette was excised from pYLsgRNA-*AcoINV3* and assembled into pYLCRISPR/Cas9Pubi-H in a 20-µL reaction containing 100 ng pYLCRISPR/Cas9Pubi-H, 50 ng pYLsgRNA-*AcoINV3*, 1 µL BsaI-HFv2, 1 µL T4 DNA ligase, and 2 µL 10× T4 DNA ligase buffer, with the same cycling conditions as above. The product was transformed into *E. coli* and selected on LB medium with 50 µg/mL kanamycin. Positive clones were identified by EcoRI restriction digestion (expected ~ 500-bp insertion) and PCR using primers VF1 (5′-GATCTAGTAACATAGATGACACCGCG-3′) and OsU3-R (5′-GCGGTAGCCAATTATACCTT-3′), yielding a ~ 350–400 bp product. The empty vector yielded no PCR product. The verified plasmid was designated pYLCRISPR/Cas9Pubi-H-*AcoINV3*.

### Development of pineapple *AcoINV3* mutants

The verified plasmid pYLCRISPR/Cas9Pubi-H-*AcoINV3* was introduced into *Agrobacterium tumefaciens* EHA105 competent cells by electroporation. Embryogenic callus was induced and embryogenic cell suspension (ECS) was established as described previously [25]. ECS at late exponential phase was resuspended by repeated pipetting, and 1 mL packed cell volume (PCV) was transferred to 20 mL fresh medium in a 100-mL conical flask. The culture was shaken at 80 rpm at 24 °C under a 16-h light/8-h dark photoperiod until the late exponential phase. The ECS was allowed to settle for 30 min, and the supernatant was decanted. The prepared *A. tumefaciens* working solution was added for 10-min infection, followed by centrifugation at 1000 rpm for 5 min. The pellet was gently shaken at < 100 rpm for 5 min, the bacterial solution was decanted, and a small volume of resuspension medium was added. The material was transferred using a cut 1-mL pipette tip onto sterile filter paper overlaid on absorbent paper to remove excess liquid. The EC material with filter paper was then transferred onto co-cultivation medium M1 (1/2 MS, 1.16 mg/L 2,4-D, 0.23 mg/L ZT, 30 g/L sucrose, 100–200 µM AS, 2.5 g/L Gelrite, pH 5.4) and cultured in darkness at 28 °C for 3 days. The material was transferred to selection medium M2 (M1 composition plus 20 mg/L kanamycin, pH 5.4) for 2 weeks, then to somatic embryo induction medium M3 (M2 plus 500 mg/L carbenicillin, pH 5.8) under light for 1 week. Subsequently, the material was transferred to regeneration medium RD1 (1/2 MS, 3 mg/L TDZ, 20 mg/L kanamycin, 500 mg/L carbenicillin, 30 g/L sucrose, 2.5 g/L Gelrite, pH 5.8) under light for 4 weeks, followed by RD2 (1/2 MS, 0.5 mg/L TDZ, 0.4 mg/L IBA, 20 mg/L kanamycin, 500 mg/L carbenicillin, 30 g/L sucrose, 2.5 g/L Gelrite, pH 5.8) under light for 5 weeks. The material was then transferred to germination medium REG (1/2 MS, 0.23 mg/L 6-BA, 0.175 mg/L IAA, 20 mg/L kanamycin, 250 mg/L carbenicillin, 30 g/L sucrose, 2.5 g/L Gelrite, pH 5.8) under light until resistant shoots emerged. Shoots approximately 5 cm in length were subjected to root induction, and plantlets with well-developed root systems were acclimatized for 4–6 weeks before transplantation. Genomic DNA was extracted from transgenic plants, and the target region was amplified using primers P1 (5′-tgatccaactcatcccacac-3′) and P2 (5′-gtagttcgacgactcgagag-3′). The PCR products were sequenced to identify the types and frequencies of insertions and deletions.

### Vector construction for pineapple *AcoINV3*-CSE-knockout mutant

The base vector pH-ePPE was obtained from Addgene (#183097) [26]. Because the 5′-CCGAC-3′ target lies in the standard SpCas9 prime-editing window, a 9-bp deletion strategy encompassing this sequence was adopted. The target deletion sequence was 5′-ccgacaaac-3′. Complementary oligonucleotides Oligo C (5′-CTTG AATATCCGACAAACTTAGGGTTTAAGAGCTATGCTGGAAACAGCATAGCAAGTTGAAATAAGGCTAGTCCGTTATCAACTTGAAAAAGTGGCACCGAGTCGGTGCTTTTTTAATATCTTAGGGAGGCCATAACTTCTAAAACC-3′) and Oligo D (5′- GGTTTTAGAAGTTATGGCCTCCCTAAGATATTAAAAAAGCACCGACTCGGTGCCACTTTTTCAAGTTGATAACGGACTAGCCTTATTTCAACTTGCTATGCTGTTTCCAGCATAGCTCTTAAACCCTAAGTTTGTCGGATATTCAAG-3′) were synthesized and PAGE-purified. Oligo C contains 5′-CTTG and 3′-AACC overhangs compatible with the BsaI-generated sticky ends (CAAG and GGTT, respectively) in the vector. For annealing, equimolar amounts of Oligo C and Oligo D (100 µM each) were combined, heated at 95 °C for 5 min, and slowly cooled to 25 °C at 0.1 °C/min. The pH-ePPE plasmid (1 µg) was digested with BsaI-HFv2 at 37 °C for 1–2 h, and the ~ 18,000-bp vector backbone was gel-purified after excision of the ~ 1,600-bp intervening fragment between the two BsaI sites. The purified backbone was adjusted to 30 ng/µL. The annealed oligonucleotide (10-fold diluted) was ligated into the BsaI-digested pH-ePPE backbone in a 20-µL Golden Gate reaction containing 50–100 ng vector, 1 µL diluted oligo, 1 µL BsaI-HFv2, 1 µL T4 DNA ligase, and 2 µL 10× T4 DNA ligase buffer. The reaction was subjected to 20 cycles of 37 °C/5 min and 16 °C/10 min, followed by 37 °C/30 min and 80 °C/10 min. The product was transformed into *E. coli* DH5α competent cells and selected on LB agar containing 50 µg/mL kanamycin. Positive clones were identified by colony PCR using primers Det-F (5′-GATCCTGCAGGCATGCAAGCTT-3′) and Ubi-90R (5′-CGTCGTTTTACAACGTCGTGACTGGG-3′), yielding an expected ~ 500-bp product; negative clones showed no amplification. Plasmids from positive clones were extracted and sequenced using the OsU3 promoter universal primer U3cef (5′-TGTGCAGATGATCCGTGGC-3′) to confirm correct pegRNA insertion. The verified plasmid was designated pePPE/INV3.

### Developing pineapple *AcoINV3*-CSE-knockout mutants

The verified construct pePPE/INV3 was introduced into *Agrobacterium tumefaciens* EHA105 electrocompetent cells by electroporation. Transformants were selected on LB plates containing 50 µg/mL kanamycin and 20 µg/mL rifampicin at 28 °C for 2–3 days. Plant transformation was performed as described in “developing pineapple *AcoINV3* mutant”. Genomic DNA was extracted from transgenic plants, and PCR was performed using primers P3 (5′- gtatcaatttagtgcccggt-3′) and P4 (5′-agtgtgggatgagttggatc-3′). The wild-type allele produced a 405 bp amplicon, whereas the 9 bp deletion mutant yielded a 396 bp band. The PCR products were subjected to direct Sanger sequencing for final confirmation.

### Transcription activity measurement

The plasmid pSB167-pL2_pSB90_2 × 35 S::fLUC-I::tNOS_tMAS::rLUC-I::pMAS [27] was obtained from Addgene (#123200). P_INV3_ and P_INV3−CSE_ were synthesized and cloned into pSB167-pL2_pSB90_2 × 35 S::fLUC-I::tNOS_tMAS::rLUC-I::pMAS respectively. The plasmid was transformed into *Agrobacterium* EHA105. The transformed *Agrobacterium* was infiltrated into tobacco leaves (*Nicotiana benthamiana*). After propagation for 48 h under 25℃, tobacco plants were transferred into a 15℃ incubator for 0.5, 1, 2, 4, 8 h, respectively. LUC activity was measured according to the method published [28]. Firefly Renilla (REN) gene driven by CaMV 35 S promoter (35Spro: REN) was used as an internal control. Transcriptional activity was calculated by the ratio of LUC to REN.

### Prediction of off-target sites

Possible off-target sites were predicted using Cas-OFFinder according to the published paper [29]. The target sequences were entered into the ‘Query Sequences’ box to identify potential off-target loci (http://www.rgenome.net/cas-offinder/). Primers flanking these sites were designed for PCR amplification and sequencing. The resulting sequences were aligned with the pineapple reference genome to identify off-target mutations.

### Translucency incidence determination

Translucency incidence was calculated after the pineapple fruits were collected. For each treatment, 100 fruits were chosen randomly. The fruits were cut longitudinally into two halves. If there were water stains in the section, this fruit was considered as translucent. Each treatment consisted of four replicates. The significant difference between treatments was tested by analysis of variance (ANOVA) and significant difference was separated using Duncan’s multiple range test (DMRT). SPSS software was used to determine significant difference at *p* ≤ 0.05.

### QRT-PCR experiment

QRT-PCR was performed in strict accordance with the MIQE guidelines. Total RNA was extracted using TRIzol Reagent (Thermo Fisher Scientific), and its purity (OD₂₆₀/₂₈₀ = 1.90–2.05) and integrity were verified prior to reverse transcription. First-strand cDNA was synthesized from 1 µg of total RNA using the RevertAid RT Kit (Thermo Fisher Scientific) with oligo(dT)₁₈ primers. The resulting cDNA was diluted 1:5 with nuclease-free water and used as the template. Real-time PCR was conducted on an ABI QuantStudio™ 5 System (Thermo Fisher Scientific) using SYBR Premix Ex Taq II (Tli RNaseH Plus) (Takara, RR820A). Each 20 µL reaction contained 10 µL SYBR Premix Ex Taq II, 0.8 µL each forward and reverse primer (10 µM), 2 µL diluted cDNA template, and 6.4 µL nuclease-free water. The thermal cycling conditions were: initial denaturation at 95 °C for 30 s, followed by 40 cycles of 95 °C for 5 s and 56 °C for 30 s. A melting curve analysis was performed after amplification (95 °C for 15 s, 60 °C for 1 min, ramping to 95 °C at 0.05 °C/s) to verify the specificity of amplification. No-template controls (NTC) and no-RT controls were included in each run to exclude contamination and genomic DNA amplification. Primer amplification efficiencies (E) were determined by standard curves generated from 5-fold serial dilutions (5 points) of pooled cDNA; only primers with efficiencies between 90% and 110% (R² > 0.98) were used. For the target gene *AcoINV3* (GenBank: XM_020255359.1), the primer sequences were *AcoINV3*-F, 5′-CAGTTGGCGGCATTAATGCT-3′ and *AcoINV3*-R, 5′-CTTAAAATCCCGGCTCCTGT-3′. The housekeeping genes *AcoTubulin* (GenBank: XM_020225526.1) and *AcoActin* (GenBank: XM_020251131.1) were selected as the reference genes for qRT-PCR normalization. To validate its expression stability under the present experimental conditions, the raw Ct values of *AcoTubulin* and *AcoActin* were analyzed across all biological samples. The Ct values of *AcoTubulin* ranged from 18.4 to 20.3, with a mean of 19.35 ± 0.95. The coefficient of variation (CV) was 2.7%. The Ct values of *AcoActin* ranged from 17.7 to 19.4, with a mean of 18.55 ± 0.85. The coefficient of variation (CV) was 2.4%. No significant difference in *AcoTubulin* expression or *AcoActin* expression was detected among experimental groups (one-way ANOVA, *P* = 0.86 and 0.89 respectively). The amplification efficiency of *AcoTubulin* primers was determined by standard curve analysis using five-fold serial dilutions of cDNA, yielding an efficiency of 98.2% and a determination coefficient (R²) of 0.997. The corresponding values for *AcoActin* were 97.1% and 0.993 respectively. The values for *AcoINV3* were 97.4% and 0.994 respectively. A single peak was observed in the melting curve analysis. *AcoTubulin* and *AcoActin* were deemed suitable as the reference genes for relative quantification in this study. All reactions were run in technical triplicate for each of the four biological replicates (*n* = 4). Relative expression levels were calculated using the 2^−ΔΔCt^ method and normalized to *AcoTubulin* (GenBank: XM_020225526.1). The primer sequences for amplifying *AcoTubulin* were AcoTubulin-F, 5′-gagctctactgtctcgaaca-3′ and AcoTubulin-R, 5′-aaaacgtgttgaacgcgtcg-3′. The primer sequences for amplifying *AcoActin* were AcoActin-F, 5′-cgacaatggaactggaatgg-3′ and AcoActin-R, 5′- ctgacaatgccgtgctcaat-3′. No amplification was detected in NTC and no-RT controls, excluding contamination and genomic DNA interference.

### Statistical analysis

Four replicate units were established per treatment. Differences among treatments were assessed via ANOVA, with post-hoc separation of means by DMRT. Significance was declared at the 0.05 probability level using SPSS. Values are reported as mean ± standard error.

## Results

### Pineapple translucency induced by 15℃ treatment during fruit enlargement was associated with elevated fructose and glucose levels in pulp intercellular spaces

**Fig. 1:**
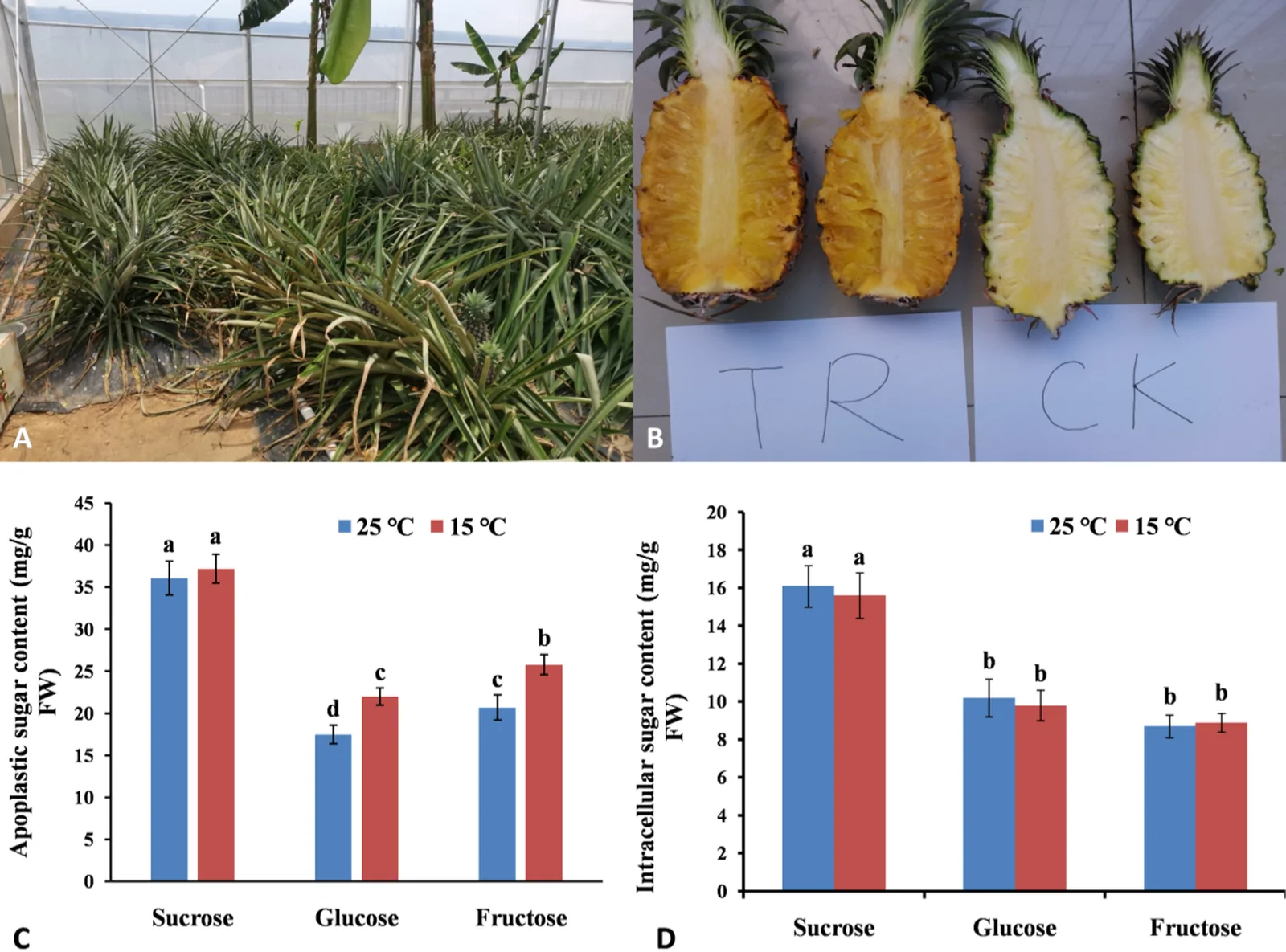
Sugar content in pineapple fruit. **A** shows pineapple plants grown in a greenhouse. **B** shows translucent (TR) and normal (CK) pineapple fruits. The standard A4 paper (210 mm × 297 mm) placed beneath the samples serves as a size reference. **C** shows apoplastic sugar content in pineapple fruit. **D** shows intracellular sugar content in pineapple fruit. 25 ℃ represents the pulp of pineapples grown continuously in a 25 ℃ greenhouse; 15 ℃ represents the pulp of pineapple plants placed in a 15 ℃ greenhouse for 30 days during the fruit enlargement period and then transferred to a 25 ℃ greenhouse until maturity. Different lowercase letters indicate significant differences at *p* ≤ 0.05 using SPSS software. Four independent experiments were conducted for each treatment. Each independent experiment consists of four biological replicates for each sample. The significant difference between treatments was tested by ANOVA and significant difference was separated using DMRT

To identify the relationship among carbohydrate metabolism, chilling and flesh translucency, sugar contents in pulp were measured after pineapple plants in fruit enlargement stage were placed in a 15 ℃ greenhouse for 30 days (Fig. 1A and B). Results showed that the fructose and glucose contents in pulp intercellular space were significantly higher than those of pineapple plants grown continuously in a 25 ℃ greenhouse, while the sucrose content in pulp intercellular space was similar with those of pineapple plants continuously in 25 ℃ (Fig. 1C). The intracellular sucrose, glucose, and fructose content showed no significant difference compared to the control (Fig. 1D). Pineapple plants grown in 15 ℃ greenhouse had larger fruits (1225 ± 41 g) than those grown in 25 ℃ greenhouse (1020 ± 40 g). Pineapple plants grown in 15 ℃ greenhouse had higher translucency incidence (46%) than those grown in 25 ℃ greenhouse (12%). These demonstrated that the formation of pineapple translucency and fruit expansion can be induced by 15℃ treatment during fruit enlargement stage. These inducements might be due to the increase of fructose and glucose in intercellular space of pineapple pulp. Decreasing the fructose and glucose contents in pulp intercellular space may reduce the translucency incidence of pineapple.

### Both translucency incidence and single-fruit weight of pineapple *AcoINV3* mutant were less than those of control

**Fig. 2:**
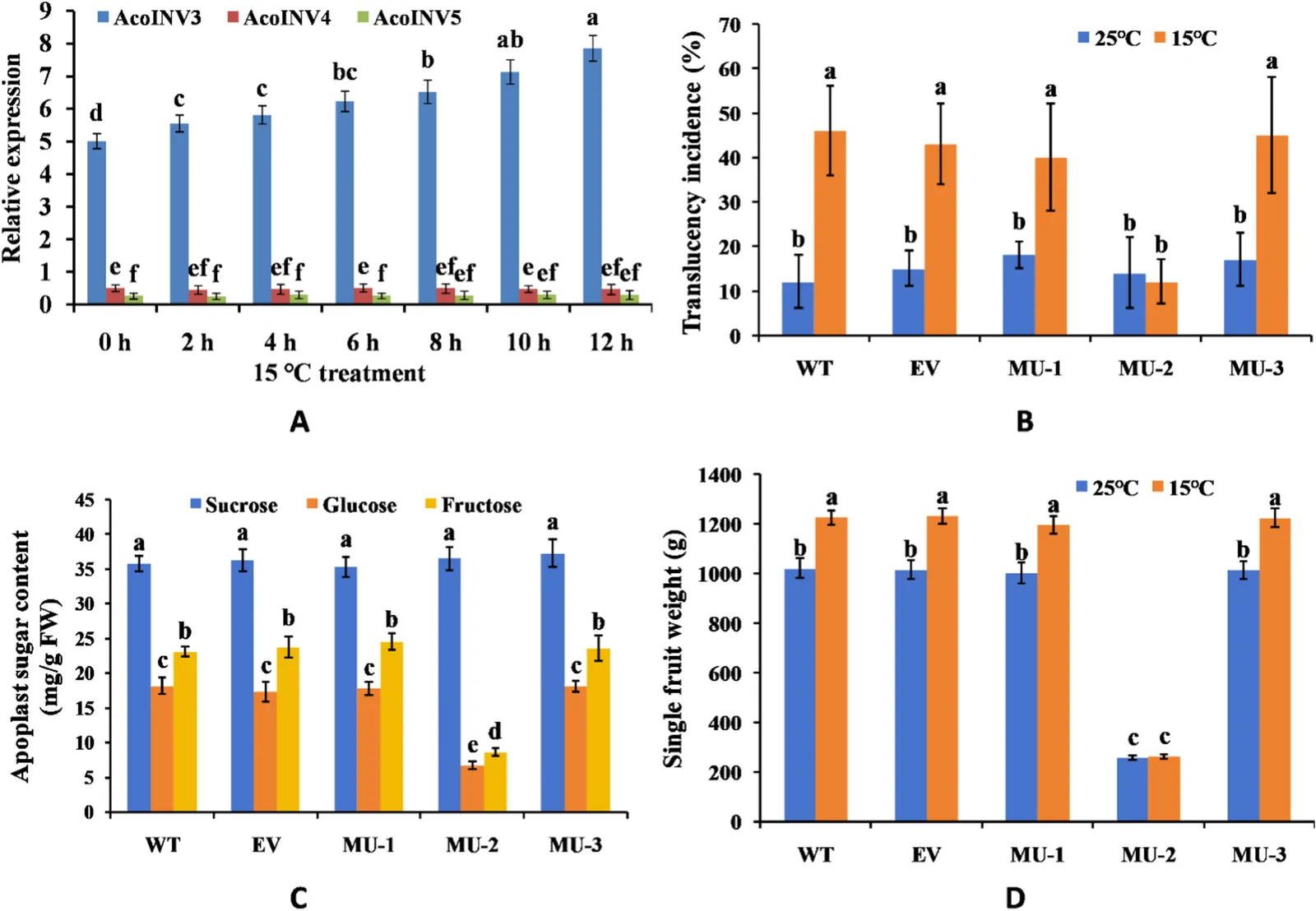
Some traits of pineapple *AcoINV3* mutants. **A** referred to relative expression of *AcoINV3*, *AcoINV4* and *AcoINV5* in fruit of TN17 plants after they were treated with 15℃. 0–12 h represented that the pineapple plants were put in 15 ℃ incubator for 0–12 h respectively during fruit enlargement. Relative expression of *AcoINV3* in pulp was measured. **B** referred to apoplast sugar content in pineapple pulp. **C** referred to translucency incidence of pineapple lines. **D** referred to single fruit weight of pineapple lines. WT referred to wild-type TN17 pineapple. EV referred to TN17 pineapple transformed with empty vector. Different lowercase letters indicate significant differences at *p* ≤ 0.05 using SPSS software. Four independent experiments were conducted for each treatment. Each independent experiment consists of four biological replicates for each sample. The significant difference between treatments was tested by ANOVA and significant difference was separated using DMRT

There are 14 homologous sequences of invertase genes in pineapple genome, among which *AcoINV1* (XM_020257109.1), *AcoINV2* (XM_020228131.1), *AcoINV3* (XM_020255359.1), *AcoINV4* (XM_020255089.1) and *AcoINV5* (XM_020255088.1) encode acid invertases. *AcoINV3*, *AcoINV4* and *AcoINV5* encode cell-wall invertases. The expressions of these three genes can be found in flower, fruit, leaf and root (Fig.S1). The expression of *AcoINV3* in fruit was much higher than those of *AcoINV4* and *AcoINV5* in fruit (Fig.S1). Similar results have also been found in flower (Fig.S1). The expression level of *AcoINV3* in fruits treated with 15℃ during fruit enlargement period was significantly higher than that of the control (Fig. 2A). After the plants were put in 15 ℃ incubator for 0, 2, 4, 6, 8, 10 and 12 h respectively, the expression level of *AcoINV3* in fruit increased with the duration of 15℃ treatment (Fig. 2A). The expression of *AcoINV3* was up-regulated by 15℃ treatment. Neither the expression of *AcoINV4* nor that of *AcoINV5* was affected by 15℃ treatment (Fig. 2A). *AcoINV3* might be the gene responsible for catalyzing the reaction of sucrose hydrolysis in pineapple pulp intercellular space.

**Fig. 3:**
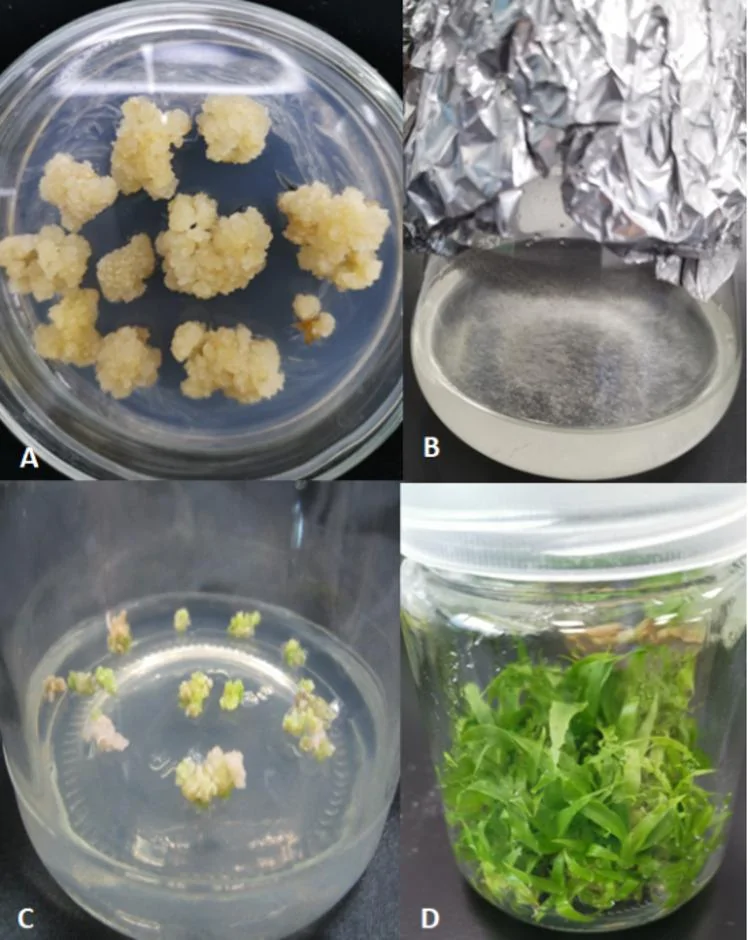
Developing pineapple *AcoINV3* mutants. **A** referred to pineapple embryogenic callus. **B** referred to pineapple embryogenic cell suspension. **C** referred to ECS cultured on differentiated medium containing 50 µg/ml kanamycin. **D** referred to differentiated seedlings cultured on medium containing 50 µg/ml kanamycin

To validate this hypothesis, pineapple *AcoINV3*-mutants were generated using the CRISPR/Cas9 system (Fig. 3A, B, C and D). Three mutants were gotten. Compared to the WT, the cDNA sequence of MU-1 has a single nucleotide substitution at position 25, where g is changed to a (ttgcggggac → ttgcgAgac). This missense mutation converts the 9th amino acid from glycine (G9) to arginine (R9) (Table S1). The remaining 582 amino acids are unchanged. In MU-2, the gg dinucleotide at positions 25–26 is replaced by at, and the a nucleotide at position 28 is deleted (Table S1). This completely disrupts the amino acid sequence from position 9 onward to bear virtually no resemblance to the WT. A stop codon is generated at nucleotide position 39, causing translation to terminate at the 13th amino acid (Table S1). MU-3 carries a 3-bp deletion spanning positions 25–27 (ttgcggggac → ttgcgac), removing the ggg codon that encodes glycine (Gly, G) (Table S1). Because the deletion is an exact multiple of three nucleotides, the reading frame remains intact. Except the single glycine residue (the 9th amino acid), the amino acid sequence is identical to the WT. No possible off-target mutation was found in MU-1, MU-2 or MU-3. They showed no significant differences in plant architecture traits compared to TN17. When plants were always grown in 25 °C greenhouse, translucency incidences of the three mutant lines and TN17 had no significant differences (Fig. 2B). If plants were put in 15 °C greenhouse for 30 days during the fruit enlargement stage, the translucency incidences of MU-1, MU-3, and TN17 were much higher than that of MU-2 (Fig. 2B). The translucency incidences of MU-2 treated with 15 °C and those always grown in 25 °C greenhouse were similar (Fig. 2B).

The fructose and glucose content in pulp intercellular space of MU-2 is significantly less than that of the pineapple transformed with the empty vector (EV) (Fig. 2C). These values of MU-2 treated with 15 ℃ were similar with those of MU-2 always grown in 25℃ greenhouse. If plants were always cultivated in 25 ℃ greenhouse, translucency incidence of MU-2 was similar with that of EV. But if they were put in 15 ℃ greenhouse for 30 days during fruit enlargement period, translucency incidence of EV (43 ± 10%) was significantly higher than that of MU-2 (11 ± 4%) (Fig. 2C). Translucency incidence of EV was similar with that of WT (Fig. 2B). Single fruit of EV treated with 15℃ was heavier than that always in 25 ℃-green house in maturity period (Fig. 2D). No matter whether the plants were treated with 15℃ or not, single-fruit weight of MU-2 was much less than that of EV in maturity period (Fig. 2D). Single-fruit weight of MU-2 treated with 15℃ was similar with that of MU-2 always grown in 25 ℃ greenhouse (Fig. 2D). Single-fruit weight of WT was similar with that of EV, no matter whether they were treated with 15℃ or not (Fig. 2D).

### The CSE in *AcoINV3* promoter had 15℃-responsive function

**Fig. 4:**
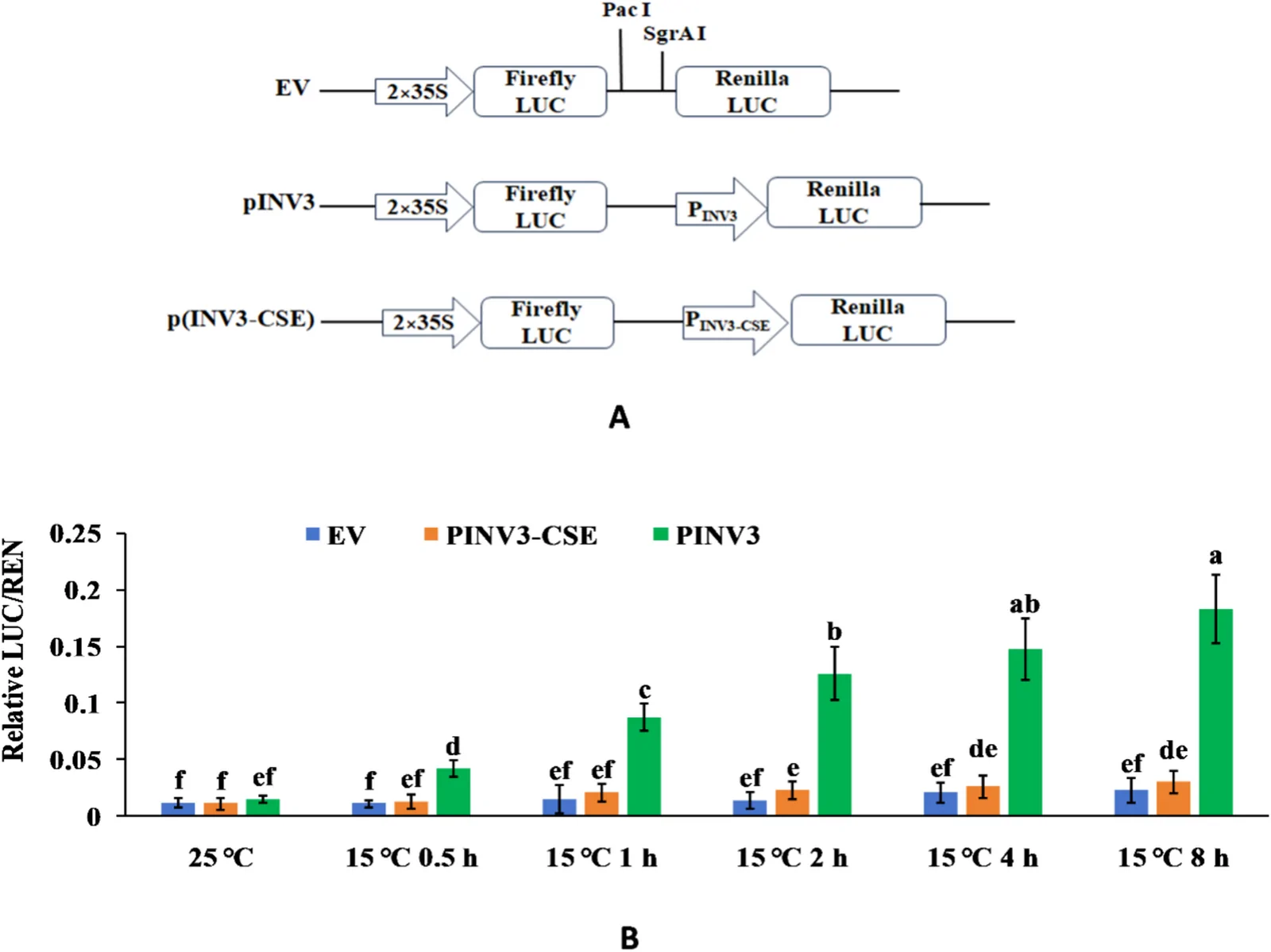
Identification the function of CSE in *AcoINV3* promoter. **A** referred to the vector map for transient transformation in tobacco leaves. **B** referred to relative LUC/REN of transient transformed tobacco leaves. EV referred to the tobacco leaves transformed with the empty vector. PINV3-CSE referred to the tobacco leaves transformed with the vector harboring the *AcoINV3* promoter in which the CSE has been deleted. PINV3 referred to the tobacco leaves transformed with the vector harboring the *AcoINV3* promoter in which the CSE was kept. 25℃ referred to the leaves of the tobacco plants always grown in a 25℃ incubator. 15℃0.5 h referred to the leaves of the tobacco plants grown in 15℃ incubator for 0.5 h. Different lowercase letters indicate significant differences at *p* ≤ 0.05 using SPSS software. Four independent experiments were conducted for each treatment. Each independent experiment consists of four biological replicates for each sample. The significant difference between treatments was tested by ANOVA and significant difference was separated using DMRT

For further studying the relationship between *AcoINV3* and 15℃ treatment, the promoter sequence of *AcoINV3* was compared with cold-shock protein (CSP) genes and stress-responsive genes in plant. A 5’-CCGAC-3’ sequence located at -445 bp upstream of the *AcoINV3* start codon was found. The 5’-CCGAC-3’ motif represents the most canonical chilling-responsive *cis*-acting element in plants, which is specifically recognized by CBF/DREB1 transcription factors [30–32]. This sequence repeatedly appears in the promoters of multiple plant-CSP genes (Table S2). It might be a CSE and have chilling responsive function. It may play critical roles when pineapple translucency and fruit weight were induced by chilling. For verifying this hypothesis, *AcoINV3* promoter without CSE was fused with dual LUCIFERASE (LUC) gene and plant-expressing vector (P_INV3−CSE_) was constructed. At the same time, the vector (P_INV3_) harboring the wild *AcoINV3* promoter containing the CSE fused with the dual LUC gene was also constructed (Fig. 4A). Tobacco leaves were transiently transformed through vacuum infiltration. The tobacco plants were put in 15 ℃ incubator for 0, 0.5, 1, 2, 4, 8 h, respectively. The fluorescence intensities of the transformed leaves were detected. Results showed that the fluorescence intensity of the leaves transformed with P_INV3−CSE_ was significantly weaker than that transformed with P_INV3_ (Fig. 4B). The fluorescence intensities of the leaves transformed with P_INV3_ were stronger and stronger with the 15℃-treatment last, while those of the leaves transformed with P_INV3−CSE_ remained weak, no matter how long time the plants were put in 15 ℃ incubator (Fig. 4B). These indicated that the expression of the gene driven by the *AcoINV3* promoter containing the CSE can be up-regulated by 15℃ treatment. The expression of the gene driven by the *AcoINV3* promoter without the CSE cannot be regulated by 15℃ treatment. The CSE in *AcoINV3* promoter had 15℃-responsive function.

### Knockout of the CSE in *AcoINV3* promoter reduced translucency incidence without affecting single-fruit weight under 15 °C-condition

**Fig. 5:**
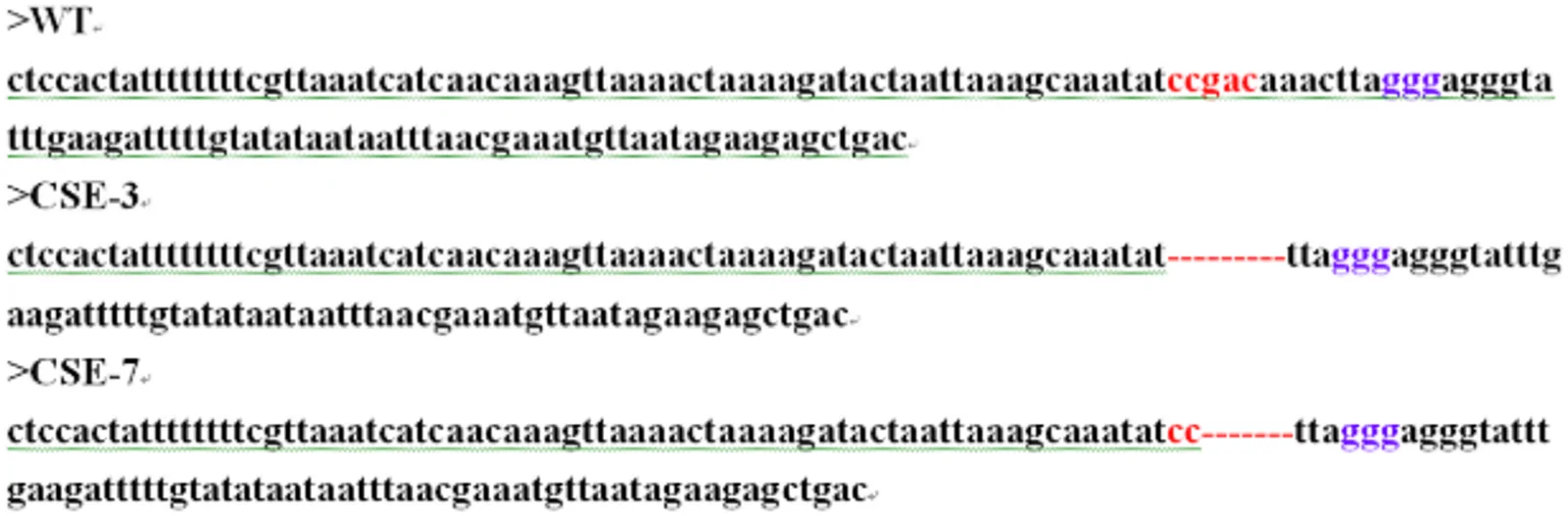
Target sequences of the CSE-edited lines. PAM sites were shown in blue lowercase letters. CSE sequence was shown in red letters. Deleted bases were shown in dash lines

**Fig. 6:**
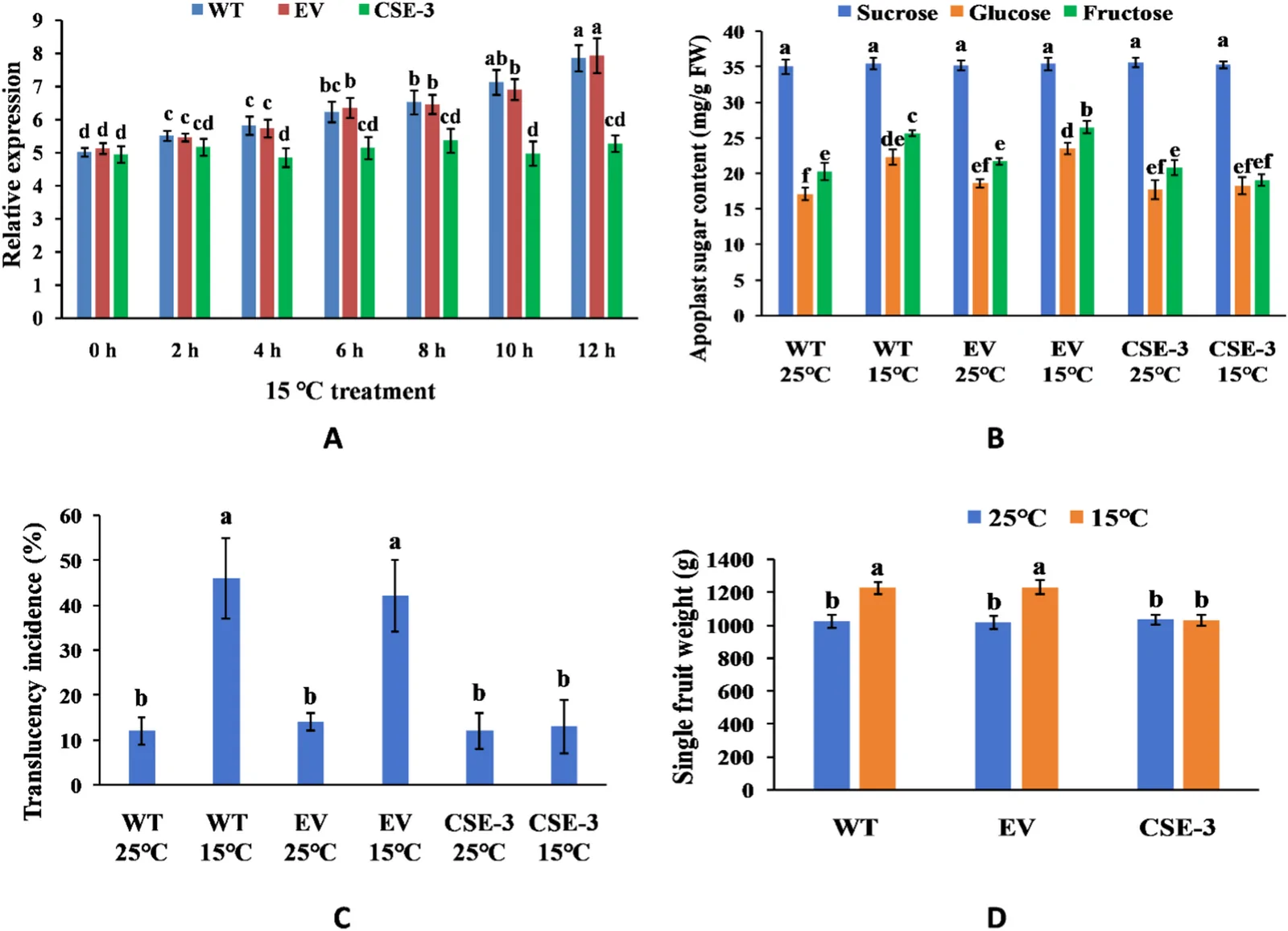
Some phenotypes of the pineapple CSE-edited line. **A** showed expression levels of *AcoINV3* in fruits of pineapple plants subjected to 15℃ treatment during the fruit enlargement period. **B** referred to apoplast sugar content in pineapple pulp. **C** referred to translucency incidence of pineapple lines. **D** referred to single fruit weight of pineapple lines. 25℃ indicated the pulp from plants grown continuously in a 25℃ greenhouse; 15℃ indicated the pulp from the plants transferred to a 15℃ incubator for 30 days during fruit enlargement period and then moved to a 25℃ greenhouse until maturity. WT referred to the wild-type TN17 pineapple. EV referred to the TN17 transformed with the empty vector. Different lowercase letters indicate significant differences at *p* ≤ 0.05 using SPSS software. Four independent experiments were conducted for each treatment. Each independent experiment consists of four biological replicates for each sample. The significant difference between treatments was tested by ANOVA and significant difference was separated using DMRT

For further investigating the function of the CSE in *AcoINV3* promoter, this element was knocked out using prime edition, generating twenty transgenic lines designated CSE-1 through CSE-20. Editing events were found in CSE-3 and CSE-7, with CSE-3 showing complete knockout and CSE-7 showing partial knockout of the CSE (Fig. 5). No off-target mutation was found in CSE-3 or CSE-7. CSE-3 was chosen for subsequent experiments. During the fruit enlargement period, pineapple plants were transferred to a 15 ℃ greenhouse for 0, 2, 4, 6, 8, and 10 days respectively, and *AcoINV3* expression levels in the fruit pulp were analyzed. Under constant 25 °C conditions, *AcoINV3* expression in CSE-3 fruits was comparable to that in the control fruits transformed with the empty vector (EV). However, upon exposure to 15 °C, *AcoINV3* expression in EV fruits was significantly higher than that in CSE-3 fruits. Notably, *AcoINV3* expression in CSE-3 fruits under 15℃ treatment remained comparable to that in plants grown continuously at 25 °C (Fig. 6A). These demonstrated that the CSE was associated with the 15℃-induced expression of *AcoINV3*.

Fructose and glucose contents in pulp apoplast were up-regulated by 15℃ treatment during the fruit enlargement period. For determining whether this up-regulation was mediated by the CSE within the *AcoINV3* promoter, sugar contents in pulp were quantified. Sucrose levels in the pulp apoplast remained similar among WT, EV and CSE-3 under both 25℃ and 15℃ conditions (Fig. 6B). The glucose and fructose contents in WT and EV pulp apoplast were significantly higher at 15 °C than at 25 °C. The glucose and fructose contents in CSE-3 pulp apoplast under 15℃ were comparable to those at 25 °C (Fig. 6B). These demonstrated that the CSE in *AcoINV3* promoter was associated with the 15℃-induced up-regulation of fructose and glucose in the pulp apoplast during fruit expansion.

Translucency incidence was assessed at fruit maturity. While CSE-3 and EV fruits exhibited comparable translucency under continuous 25 °C growth, the translucency incidence of CSE-3 treated with 15 °C for 30 days during fruit enlargement was significantly lower than that of EV treated with 15℃ (Fig. 6C). 15℃ treatment did not affect translucency in CSE-3 but substantially elevated it in EV (Fig. 6C). Knockout of the CSE in *AcoINV3* promoter mitigated 15℃-induced translucency during the fruit enlargement period.

For investigating the effect of knockout the CSE in *AcoINV3* promoter on fruit growth, single-fruit weight was measured at maturity. No difference was observed among CSE-3 plants exposed to 15 °C, CSE-3 plants continuously at 25 °C, and EV plants remained at 25 °C (Fig. 6D). In contrast, EV plants subjected to 15 °C for 30 days during fruit enlargement produced heavier fruits than those grown at 25 °C. WT and EV fruits exhibited comparable weights under the same temperature conditions, indicating the transformation procedure itself did not affect fruit growth. These demonstrated that 15℃ treatment during fruit enlargement promoted fruit weight in a CSE-dependent manner. Knockout of this element abolished the 15℃-induced weight increase. The CSE in *AcoINV3* promoter was associated with the up-regulation of fruit weight by 15℃ treatment. Knockout of the CSE in *AcoINV3* promoter reduced translucency incidence without affecting single-fruit weight under 15 °C-condition (Fig. 6C and D).

## Discussion

Plant genomes contain numerous CSP genes, whose expression is regulated by cold-shock transcription factors (CSFs) [33–35]. The promoters of CSP genes always contain CSEs [29–31, 36, 37]. Under chilling conditions, CSFs bind to CSEs, inducing CSP gene expression [38, 39]. The CSE (CCGAC) motifs have been identified in the promoters of many CSP genes in *Arabidopsis thaliana*, *Triticum aestivum* L., *Zea mays* L., and *Oryza sativa* L [40–43]. Similar CSEs (CACATG, CACGTG) have also been found in the promoter of banana CSP gene *MaNAC1* [44]. After analyzing in cold-sensitive cultivated tomato and cold-tolerant wild tomato, Guo et al. (2024) found a 60 bp InDel in the WRKY34 promoter, which determines differences in its transcription and cold tolerance among 376 tomato accessions [45]. This 60 bp fragment contains a GATA cis-regulatory element that binds to GATA29 and ATP-dependent chromatin remodeling complex SWIBs, which synergistically suppress WRKY34 expression under cold stress [45]. Overexpression of BTB-domain ubiquitin ligase adaptor SlBTB19 can enhance tomato plant sensitivity to cold stress, whereas SlBTB19 knockout mutants exhibited a cold-tolerance phenotype [46]. Under cold stress, SlWRKY2 binds to the W-box in the CRT/DRE-Binding Factor 1 (CBF1) and CBF3 promoters, directly activating their expression [46]. The transcription factor SlGRAS4 promotes chilling tolerance in tomato leaves and fruit [47]. Tomato GAI/RGA/SCR-derived family 4 SlGRAS4 forms a homodimer to self-activate its own promoter [47]. In this study, it was found that the CSE in *AcoINV3* promoter played a critical role in 15℃-treatment regulation translucency in pineapple. Regardless of whether CSE-3 plants were transferred to a 15 °C incubator, the sucrose, fructose, and glucose contents in the pulp apoplast of CSE-3 fruits remained similar to those of empty vector control fruits grown continuously at 25 °C. While fructose and glucose contents in the pulp apoplast of EV pineapple were induced by 15℃ treatment, those in CSE-3 fruits were not, suggesting that the *AcoINV3* CSE is essential for the 15℃-treatment induction of fructose and glucose accumulation in pulp apoplast. Similar results were also found in translucency incidence when pineapple plants were treated with 15℃ during fruit enlargement period. The CSE in the *AcoINV3* promoter played important roles when pineapple translucency was induced by 15℃ treatment during fruit enlargement period. In WT, MU-2 and CSE-3 fruit treated with 15 ℃ or not, sugar contents in intracellular space have also been measured. No difference has been found among them. These suggested that the sucrose hydrolysis catalyzed by AcoINV3 happened in apoplast, not in intracellular space.

Fruits are primarily composed of carbohydrates, water, and mineral elements. Over-expression of cell wall invertase enhances sink strength by promoting photosynthate translocation from source to sink organs, thereby increasing fruit weight [28]. In this study, pineapple plants treated at 15°C for 30 days produced heavier fruits than those grown continuously at 25°C, indicating that pineapple fruit weight can be induced by 15℃ treatment during fruit enlargement. This contrasts with Paull et al. (1996), who found no correlation between pre-harvest air temperature and individual fruit weight under field conditions [5]. However, their analysis relied solely on monthly temperature extremes without considering duration, which may account for the discrepancy. Consistent with our approach, Friend (1981) demonstrated that ‘Smooth Cayenne’ pineapple exposed to 15 °C nocturnal temperatures during the 2–4 months prior to harvest yielded significantly heavier fruits than those at 30 °C [48], confirming that 15℃ treatment during fruit expansion promote pineapple fruit weight. When grown continuously at 25 °C, MU-2 fruit weight was only ~ 25% of the EV control. CSE-3 fruit weight remained comparable to the 25 °C EV control regardless of 15℃ treatment. These suggested that while *AcoINV3* contributes to fruit cell division and expansion, the CSE in its promoter is important for fruit weight regulation.

**Fig. 7:**
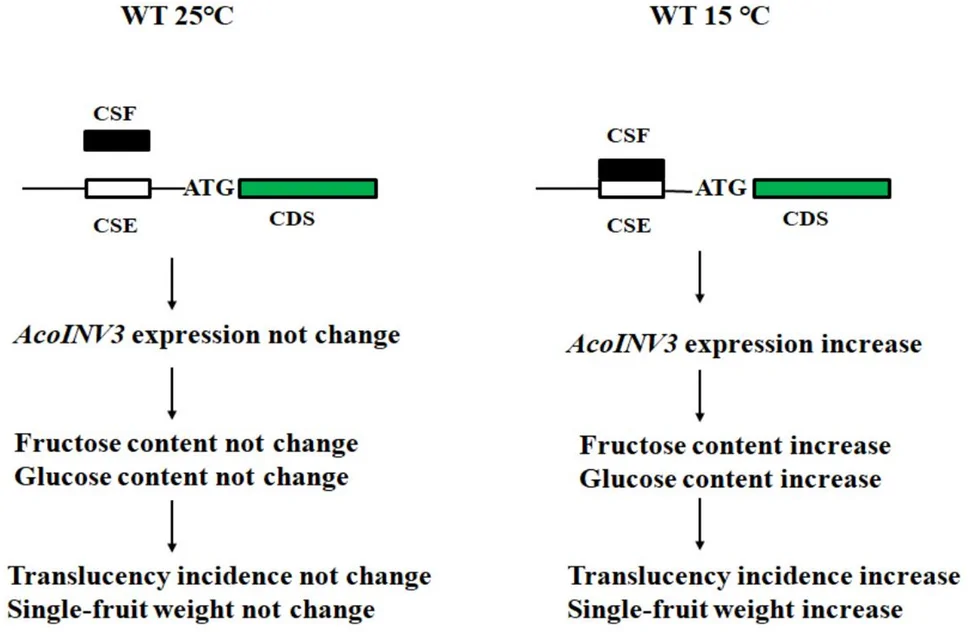
Hypothetical working model that pineapple translucency and single-fruit weight were induced by 15℃ treatment during the fruit enlargement period

Based on the findings above, a hypothetical working model was proposed explaining how 15℃ treatment during fruit enlargement regulates pineapple translucency and single-fruit weight (Fig. 7). Under normal temperatures, CSFs do not bind the CSE in the *AcoINV3* promoter, resulting in basal *AcoINV3* expression, low translucency incidence, and normal fruit weight. Under 15℃ conditions, CSFs bind to the CSE, up-regulating *AcoINV3* expression, enhancing fructose and glucose levels in the pulp, increasing single-fruit weight, promoting translucency formation. In the WT at normal temperature, the absence of CSF-CSE binding and basal *AcoINV3* expression results in normal fructose and glucose levels, normal fruit weight, and low translucency. In MU-2, a stop codon is generated at nucleotide position 39, causing translation to terminate at the 13th amino acid. The gg dinucleotide at positions 25–26 is replaced by at, and the a nucleotide at position 28 is deleted. This completely disrupts the amino acid sequence from position 9 onward to bear virtually no resemblance to the WT. Although 15℃ treatment may activate putative CSFs, *AcoINV3* has been mutated and the protein has been disrupted completely. *AcoINV3* cannot express, leading to low sugar content, reduced fruit weight, and low translucency. In CSE-3, regardless of temperature, CSFs cannot bind with cis-element in *AcoINV3* promoter, so *AcoINV3* expression remains un-induced, fructose and glucose levels stay normal, translucency incidence remains low, single-fruit weight remains unchanged. CSFs have not been identified in this study. The CSF-CSE binding mechanism remains hypothetical.

Osmotic pressure, cell turgor, and apoplastic water had close relationship with pineapple translucency [3, 7, 9]. In this study, sucrose, glucose and fructose were measured. Osmotic pressure, cell turgor, and apoplastic water content were not determined, rendering the causal link to water-soaked translucency indirect. Nevertheless, as soluble sugars are the principal osmotic determinants in pineapple fruit, alterations in sugar concentration and composition directly govern cellular osmotic potential and turgor pressure [49, 50]. The marked shifts in sugar profiles under 15℃ strongly imply corresponding changes in osmotic pressure and water potential gradients.

Measurement of cell wall invertase activity would provide definitive evidence for enzyme function [51, 52]. However, cell wall invertase and vacuolar invertase share acidic pH optima, making them difficult to discriminate in crude extracts without subcellular fractionation or isoform-specific antibodies [53–55]. Such discrimination is technically challenging in pineapple fruit owing to high phenolic content, fibrous tissue, and abundant proteolytic activity, all of which compromise enzyme stability during extraction [56, 57]. Future work will prioritize developing *AcoINV3*-specific antibodies and optimized cell wall fractionation protocols to directly quantify cell wall invertase activity and investigate potential post-translational regulatory mechanisms.

Beyond TN17, *AcoINV3* genomic sequences were amplified from Smooth Cayenne, Tainung No. 16, and Tainung No. 4. The gene is conserved across these cultivars, suggesting that the investigated molecular mechanisms may be conserved throughout the species. Although greenhouse data indicated that 15℃ treatment during fruit enlargement did not affect CSE-3 single-fruit weight, multi-location field trials across multiple cropping cycles are required to fully evaluate genotype-by-environment interactions. Such validation is currently underway.

Whole-genome sequencing provides the most comprehensive off-target detection [58–60]. However, pineapple’s high heterozygosity presents substantial technical and computational challenges that currently preclude its routine application [61–63]. Off-target effects were assessed using Cas-OFFinder combined with PCR amplification at the highest-scoring predicted off-target sites in this research. No off-target mutation was detected in any mutant line.

Cold stress typically reduces yield through freezing injury [64–66]. However, we found that constant 15 °C treatment during the fruit-enlargement period increased pineapple single-fruit weight. This temperature represents moderate chilling stress rather than freezing injury (< 0 °C), which causes ice crystal formation and reduced fruit size [67, 68]. According to our proposed model, 15 °C may activate CSFs that bind the CSE in *AcoINV3* promoter, enhancing *AcoINV3* expression and promoting sugar transfer into pulp cells, thereby increasing fruit weight. This provides a mechanistic basis for the well-established field observation that larger pineapple fruits are more prone to translucency. Field conditions introduce additional complexity: temperature fluctuations may attenuate or amplify the constant-temperature response; transient cold spells of varying duration may trigger incomplete acclimation; and cultivation management modulates source strength independently of temperature [69, 70]. Our controlled study isolates the effect of 15 °C treatment on *AcoINV3* expression, sink metabolism and translucency development, proposing a mechanism that operates within this context. Whether this effect still functions under another temperature conditions requires further experiments. A temperature gradient will be performed in following work.

The vacuum infiltration-centrifugation method employed in this study for apoplastic sugar extraction was adapted from the optimized protocol described by Lohaus et al. [71]. While this technique is widely accepted for apoplastic fluid isolation, cytosolic contamination was not directly assessed in the present study. Several precautions were taken to minimize potential contamination: (1) the centrifugation force was maintained at 950 × g, which is below the threshold at which cytoplasmic leakage becomes significant for most plant tissues [71]; (2) gentle vacuum infiltration parameters (45 mbar, 2 min, two cycles) were used to avoid mechanical damage to cells; and (3) rhamnose was included in the infiltration buffer as an internal standard to calculate the apoplast dilution factor and monitor extraction consistency. Nevertheless, the absence of direct contamination markers—such as malate dehydrogenase or glucose-6-phosphate dehydrogenase activity assays, hexose phosphate detection, or electrolyte leakage measurements—represents a limitation of this study. As noted by Lohaus et al. [71], marker enzyme activities remain low until a threshold centrifugal force is exceeded, above which these activities increase rapidly due to cytoplasmic leakage. Future studies will incorporate cytosolic marker assay to validate apoplast purity.

## Conclusions

In this study, we found that 15℃ treatment of pineapple plants during fruit enlargement increased fructose and glucose levels in the pulp apoplast and up-regulated expression of the cell wall invertase gene *AcoINV3*. Under 15℃ stress during fruit enlargement, *AcoINV3* mutant MU-2 exhibited a significantly lower incidence of translucency than empty-vector control. Its single-fruit weight was also much less than the control. A CSE in *AcoINV3* promoter was identified that functions as a 15℃-responsive cis-element. CSE-3, a CSE-knockout line of *AcoINV3*, showed significantly lower translucency rates than empty-vector-transformed plants when exposed to 15℃ treatment during fruit enlargement. The single-fruit weight of the CSE-knockout line was comparable to that of wild-type plants grown under 25℃ condition. These suggest that *AcoINV3* and its CSE-containing promoter region are associated with 15℃-responsive apoplastic hexose accumulation and translucency development under the tested condition. The CSE in the *AcoINV3* promoter has 15℃-responsive function. Targeted knockout of the CSE in *AcoINV3* promoter reduces translucency incidence without compromising single-fruit weight under 15℃ condition.

## Supplementary Information

Supplementary Material 1.

## Data Availability

The datasets used and/or analyzed during the current study are available from the corresponding author upon reasonable request.
