# Supplementary material for: A CSE-containing promoter region of AcoINV3 is associated with 15℃-induced pineapple translucency during fruit enlargement
Source: BMC Plant Biol. 2026 Aug 12;26:1915. doi: 10.1186/s12870-026-09737-7 (PMC13746609; doi:10.1186/s12870-026-09737-7)
Supplement: Supplementary file 1 [file 12870_2026_9737_MOESM1_ESM.doc]

**Supplementary file**

**Fig. S1** Relative expressions of *AcoINV3*, *AcoINV4* and *AcoINV5* in different tissues

Relative expression of *AcoINVs* in pulp was measured. Different lowercase letters indicate significant differences at p ≤ 0.05 using SPSS software. Four independent experiments were conducted for each treatment. Each independent experiment consists of four biological replicates for each sample. The significant difference between treatments was tested by ANOVA and significant difference was separated using DMRT.

**Table S1** Target sequences, cDNA sequences and amino acid sequences of *AcoINV3* in WT and mutants

Dash line showed the deleted base or deleted amino acid residue. Capital letter showed the inserted base. Red letter showed the PAM site and the premature stop codon. Blue letters showed the mutated amino acid residues. *showed the stop codon.

WT: atgggggcttcgcgcaagattgcggggacttgggtggtga

MU-1: atgggggcttcgcgcaagattgcgAggacttgggtggtga

MU-2: atgggggcttcgcgcaagattgcgATg-cttgggtggtga

MU-3: atgggggcttcgcgcaagattgcg---acttgggtggtga

>WT cDNA

atgggggcttcgcgcaagattgcggggacttgggtggtgatggtggcggtggcggtggcggtgctttcgcggtgggtcgatgtcgcggaagcgtcgcatgcggtgtacgagcatctccaatctctcgaatcgtcgaactacgatcccaagttgagaactgggtaccattttcaaccccccaagcattggatcaatgatccaaatggaccaatgtactacaatggagtctaccacctcttctaccagtacaatcctaaaggtgccgtctggggcaatattgtgtgggcccactcagtctcgaccgatctgatcaattggctagccgtcgaaccggctatccacccgactgaaccgttcgatatcaatggttgctggtctggctccgccaccatcctccctggcaaccgtccggtcattttgtacactggcgtcgacaccctcagccgtcaggtccaaaacatcgcattcccgaagaacctgtcggacccgtacctccgcaaatgggtcaagcccaaatacaacccattgatccagccagttggcggcattaatgctagctcatttcgtgaccctacaacagcatggtacggtagggacgggcgttggcggttggtgatcgggagcaagcgggggatgcgaggggaggcgatcttgtacaggagccgggattttaagcggtgggtgaaggcgaagcacgcgttgcatacggcgggcgggactggcatgtgggagtgccccgatttttacccggtgtccatcgacggcaagcaagggctcgatacgtccgagtacggggaaggggtgaagcatgtgttcaaggtgagcttggacttgacgagatacgagtactatacggttggtacgtacgaccaggaaaaagatcagtatgtgccggatggtgcatcacctgatggtaataccggtttgagatacgattacgggaacttttatgcgtcgaagacgttttacgatgccgcaggaaagagaaggatcttgtgggggtgggcgaatgagtcggactccgtgacagatgataattccaagggttgggccggcattcaggttattcctagggctgtatggctggataaagaaggaaagcaattggtgcagtggccagtggaggagattgaaactctgaggggaaagcaaattcaaatcaagcacaaaattgttaagagtggaaagttttttgaagttaaaggaattcaaactgcgcaggctgatgtggaggtgatctttgatgtgaaaagcctggagaaagctgagccctttgatccatcatggagtactaatccccagggcctatgtgggatcaagggtggagaggtgaagggtggggttgggccttttggtctttttgtcttggcctcctccaagctggaggagagaactgctgtcttcttcagggttttcaggagtgatgacaagcatgttgtcctcatgtgcaatgatcccacaaggtcgtcttataaatcaaatttgtacaaaccaacttttgcgggatttgtcgacgtcgacatcgcgaaaaatagcaagatatcgctgcggactttgattgatcactctgtggtggagagcttcggagagaacgggaaaacatgcattacatcgagagtttatccgagtttggcagtagggtcgaacgtgcatttgttcgtgttcaacaacggcgcagaggacatagaaatgtcgcagttgaagggatgggagatgaagaatcctcgcatgaatgacggagagatctag

**>**WT amino acid

MGASRKIAGTWVVMVAVAVAVLSRWVDVAEASHAVYEHLQSLESSNYDPKLRTGYHFQPPKHWINDPNGPMYYNGVYHLFYQYNPKGAVWGNIVWAHSVSTDLINWLAVEPAIHPTEPFDINGCWSGSATILPGNRPVILYTGVDTLSRQVQNIAFPKNLSDPYLRKWVKPKYNPLIQPVGGINASSFRDPTTAWYGRDGRWRLVIGSKRGMRGEAILYRSRDFKRWVKAKHALHTAGGTGMWECPDFYPVSIDGKQGLDTSEYGEGVKHVFKVSLDLTRYEYYTVGTYDQEKDQYVPDGASPDGNTGLRYDYGNFYASKTFYDAAGKRRILWGWANESDSVTDDNSKGWAGIQVIPRAVWLDKEGKQLVQWPVEEIETLRGKQIQIKHKIVKSGKFFEVKGIQTAQADVEVIFDVKSLEKAEPFDPSWSTNPQGLCGIKGGEVKGGVGPFGLFVLASSKLEERTAVFFRVFRSDDKHVVLMCNDPTRSSYKSNLYKPTFAGFVDVDIAKNSKISLRTLIDHSVVESFGENGKTCITSRVYPSLAVGSNVHLFVFNNGAEDIEMSQLKGWEMKNPRMNDGEI*

**>**MU-1 cDNA

atgggggcttcgcgcaagattgcgAggacttgggtggtgatggtggcggtggcggtggcggtgctttcgcggtgggtcgatgtcgcggaagcgtcgcatgcggtgtacgagcatctccaatctctcgaatcgtcgaactacgatcccaagttgagaactgggtaccattttcaaccccccaagcattggatcaatgatccaaatggaccaatgtactacaatggagtctaccacctcttctaccagtacaatcctaaaggtgccgtctggggcaatattgtgtgggcccactcagtctcgaccgatctgatcaattggctagccgtcgaaccggctatccacccgactgaaccgttcgatatcaatggttgctggtctggctccgccaccatcctccctggcaaccgtccggtcattttgtacactggcgtcgacaccctcagccgtcaggtccaaaacatcgcattcccgaagaacctgtcggacccgtacctccgcaaatgggtcaagcccaaatacaacccattgatccagccagttggcggcattaatgctagctcatttcgtgaccctacaacagcatggtacggtagggacgggcgttggcggttggtgatcgggagcaagcgggggatgcgaggggaggcgatcttgtacaggagccgggattttaagcggtgggtgaaggcgaagcacgcgttgcatacggcgggcgggactggcatgtgggagtgccccgatttttacccggtgtccatcgacggcaagcaagggctcgatacgtccgagtacggggaaggggtgaagcatgtgttcaaggtgagcttggacttgacgagatacgagtactatacggttggtacgtacgaccaggaaaaagatcagtatgtgccggatggtgcatcacctgatggtaataccggtttgagatacgattacgggaacttttatgcgtcgaagacgttttacgatgccgcaggaaagagaaggatcttgtgggggtgggcgaatgagtcggactccgtgacagatgataattccaagggttgggccggcattcaggttattcctagggctgtatggctggataaagaaggaaagcaattggtgcagtggccagtggaggagattgaaactctgaggggaaagcaaattcaaatcaagcacaaaattgttaagagtggaaagttttttgaagttaaaggaattcaaactgcgcaggctgatgtggaggtgatctttgatgtgaaaagcctggagaaagctgagccctttgatccatcatggagtactaatccccagggcctatgtgggatcaagggtggagaggtgaagggtggggttgggccttttggtctttttgtcttggcctcctccaagctggaggagagaactgctgtcttcttcagggttttcaggagtgatgacaagcatgttgtcctcatgtgcaatgatcccacaaggtcgtcttataaatcaaatttgtacaaaccaacttttgcgggatttgtcgacgtcgacatcgcgaaaaatagcaagatatcgctgcggactttgattgatcactctgtggtggagagcttcggagagaacgggaaaacatgcattacatcgagagtttatccgagtttggcagtagggtcgaacgtgcatttgttcgtgttcaacaacggcgcagaggacatagaaatgtcgcagttgaagggatgggagatgaagaatcctcgcatgaatgacggagagatctag

>MU-1 amino acid

MGASRKIA**R**TWVVMVAVAVAVLSRWVDVAEASHAVYEHLQSLESSNYDPKLRTGYHFQPPKHWINDPNGPMYYNGVYHLFYQYNPKGAVWGNIVWAHSVSTDLINWLAVEPAIHPTEPFDINGCWSGSATILPGNRPVILYTGVDTLSRQVQNIAFPKNLSDPYLRKWVKPKYNPLIQPVGGINASSFRDPTTAWYGRDGRWRLVIGSKRGMRGEAILYRSRDFKRWVKAKHALHTAGGTGMWECPDFYPVSIDGKQGLDTSEYGEGVKHVFKVSLDLTRYEYYTVGTYDQEKDQYVPDGASPDGNTGLRYDYGNFYASKTFYDAAGKRRILWGWANESDSVTDDNSKGWAGIQVIPRAVWLDKEGKQLVQWPVEEIETLRGKQIQIKHKIVKSGKFFEVKGIQTAQADVEVIFDVKSLEKAEPFDPSWSTNPQGLCGIKGGEVKGGVGPFGLFVLASSKLEERTAVFFRVFRSDDKHVVLMCNDPTRSSYKSNLYKPTFAGFVDVDIAKNSKISLRTLIDHSVVESFGENGKTCITSRVYPSLAVGSNVHLFVFNNGAEDIEMSQLKGWEMKNPRMNDGEI*

**>**MU-2 cDNA

atgggggcttcgcgcaagattgcgATg-cttgggtggtgatggtggcggtggcggtggcggtgctttcgcggtgggtcgatgtcgcggaagcgtcgcatgcggtgtacgagcatctccaatctctcgaatcgtcgaactacgatcccaagttgagaactgggtaccattttcaaccccccaagcattggatcaatgatccaaatggaccaatgtactacaatggagtctaccacctcttctaccagtacaatcctaaaggtgccgtctggggcaatattgtgtgggcccactcagtctcgaccgatctgatcaattggctagccgtcgaaccggctatccacccgactgaaccgttcgatatcaatggttgctggtctggctccgccaccatcctccctggcaaccgtccggtcattttgtacactggcgtcgacaccctcagccgtcaggtccaaaacatcgcattcccgaagaacctgtcggacccgtacctccgcaaatgggtcaagcccaaatacaacccattgatccagccagttggcggcattaatgctagctcatttcgtgaccctacaacagcatggtacggtagggacgggcgttggcggttggtgatcgggagcaagcgggggatgcgaggggaggcgatcttgtacaggagccgggattttaagcggtgggtgaaggcgaagcacgcgttgcatacggcgggcgggactggcatgtgggagtgccccgatttttacccggtgtccatcgacggcaagcaagggctcgatacgtccgagtacggggaaggggtgaagcatgtgttcaaggtgagcttggacttgacgagatacgagtactatacggttggtacgtacgaccaggaaaaagatcagtatgtgccggatggtgcatcacctgatggtaataccggtttgagatacgattacgggaacttttatgcgtcgaagacgttttacgatgccgcaggaaagagaaggatcttgtgggggtgggcgaatgagtcggactccgtgacagatgataattccaagggttgggccggcattcaggttattcctagggctgtatggctggataaagaaggaaagcaattggtgcagtggccagtggaggagattgaaactctgaggggaaagcaaattcaaatcaagcacaaaattgttaagagtggaaagttttttgaagttaaaggaattcaaactgcgcaggctgatgtggaggtgatctttgatgtgaaaagcctggagaaagctgagccctttgatccatcatggagtactaatccccagggcctatgtgggatcaagggtggagaggtgaagggtggggttgggccttttggtctttttgtcttggcctcctccaagctggaggagagaactgctgtcttcttcagggttttcaggagtgatgacaagcatgttgtcctcatgtgcaatgatcccacaaggtcgtcttataaatcaaatttgtacaaaccaacttttgcgggatttgtcgacgtcgacatcgcgaaaaatagcaagatatcgctgcggactttgattgatcactctgtggtggagagcttcggagagaacgggaaaacatgcattacatcgagagtttatccgagtttggcagtagggtcgaacgtgcatttgttcgtgttcaacaacggcgcagaggacatagaaatgtcgcagttgaagggatgggagatgaagaatcctcgcatgaatgacggagagatctag

**>**MU-2 amino acid

MGASRKIAMLGW*WWRWRWRCFRGGSMSRKRRMRCTSISNLSNRRTTIPS*ELGTIFNPPSIGSMIQMDQCTTMESTTSSTSTILKVPSGAILCGPTQSRPI*SIG*PSNRLSTRLNRSISMVAGLAPPPSSLATVRSFCTLASTPSAVRSKTSHSRRTCRTRTSANGSSPNTTH*SSQLAALMLAHFVTLQQHGTVGTGVGGW*SGASGGCEGRRSCTGAGILSGG*RRSTRCIRRAGLACGSAPIFTRCPSTASKGSIRPSTGKG*SMCSR*AWT*RDTSTIRLVRTTRKKISMCRMVHHLMVIPV*DTITGTFMRRRRFTMPQEREGSCGGGRMSRTP*QMIIPRVGPAFRLFLGLYGWIKKESNWCSGQWRRLKL*GESKFKSSTKLLRVESFLKLKEFKLRRLMWR*SLM*KAWRKLSPLIHHGVLIPRAYVGSRVER*RVGLGLLVFLSWPPPSWRRELLSSSGFSGVMTSMLSSCAMIPQGRLINQICTNQLLRDLSTSTSRKIARYRCGL*LITLWWRASERTGKHALHREFIRVWQ*GRTCICSCSTTAQRT*KCRS*RDGR*RILA*MTERS

**>**MU-3 cDNA

atgggggcttcgcgcaagattgcg---acttgggtggtgatggtggcggtggcggtggcggtgctttcgcggtgggtcgatgtcgcggaagcgtcgcatgcggtgtacgagcatctccaatctctcgaatcgtcgaactacgatcccaagttgagaactgggtaccattttcaaccccccaagcattggatcaatgatccaaatggaccaatgtactacaatggagtctaccacctcttctaccagtacaatcctaaaggtgccgtctggggcaatattgtgtgggcccactcagtctcgaccgatctgatcaattggctagccgtcgaaccggctatccacccgactgaaccgttcgatatcaatggttgctggtctggctccgccaccatcctccctggcaaccgtccggtcattttgtacactggcgtcgacaccctcagccgtcaggtccaaaacatcgcattcccgaagaacctgtcggacccgtacctccgcaaatgggtcaagcccaaatacaacccattgatccagccagttggcggcattaatgctagctcatttcgtgaccctacaacagcatggtacggtagggacgggcgttggcggttggtgatcgggagcaagcgggggatgcgaggggaggcgatcttgtacaggagccgggattttaagcggtgggtgaaggcgaagcacgcgttgcatacggcgggcgggactggcatgtgggagtgccccgatttttacccggtgtccatcgacggcaagcaagggctcgatacgtccgagtacggggaaggggtgaagcatgtgttcaaggtgagcttggacttgacgagatacgagtactatacggttggtacgtacgaccaggaaaaagatcagtatgtgccggatggtgcatcacctgatggtaataccggtttgagatacgattacgggaacttttatgcgtcgaagacgttttacgatgccgcaggaaagagaaggatcttgtgggggtgggcgaatgagtcggactccgtgacagatgataattccaagggttgggccggcattcaggttattcctagggctgtatggctggataaagaaggaaagcaattggtgcagtggccagtggaggagattgaaactctgaggggaaagcaaattcaaatcaagcacaaaattgttaagagtggaaagttttttgaagttaaaggaattcaaactgcgcaggctgatgtggaggtgatctttgatgtgaaaagcctggagaaagctgagccctttgatccatcatggagtactaatccccagggcctatgtgggatcaagggtggagaggtgaagggtggggttgggccttttggtctttttgtcttggcctcctccaagctggaggagagaactgctgtcttcttcagggttttcaggagtgatgacaagcatgttgtcctcatgtgcaatgatcccacaaggtcgtcttataaatcaaatttgtacaaaccaacttttgcgggatttgtcgacgtcgacatcgcgaaaaatagcaagatatcgctgcggactttgattgatcactctgtggtggagagcttcggagagaacgggaaaacatgcattacatcgagagtttatccgagtttggcagtagggtcgaacgtgcatttgttcgtgttcaacaacggcgcagaggacatagaaatgtcgcagttgaagggatgggagatgaagaatcctcgcatgaatgacggagagatctag

**>**MU-3 amino acid

MGASRKIA-TWVVMVAVAVAVLSRWVDVAEASHAVYEHLQSLESSNYDPKLRTGYHFQPPKHWINDPNGPMYYNGVYHLFYQYNPKGAVWGNIVWAHSVSTDLINWLAVEPAIHPTEPFDINGCWSGSATILPGNRPVILYTGVDTLSRQVQNIAFPKNLSDPYLRKWVKPKYNPLIQPVGGINASSFRDPTTAWYGRDGRWRLVIGSKRGMRGEAILYRSRDFKRWVKAKHALHTAGGTGMWECPDFYPVSIDGKQGLDTSEYGEGVKHVFKVSLDLTRYEYYTVGTYDQEKDQYVPDGASPDGNTGLRYDYGNFYASKTFYDAAGKRRILWGWANESDSVTDDNSKGWAGIQVIPRAVWLDKEGKQLVQWPVEEIETLRGKQIQIKHKIVKSGKFFEVKGIQTAQADVEVIFDVKSLEKAEPFDPSWSTNPQGLCGIKGGEVKGGVGPFGLFVLASSKLEERTAVFFRVFRSDDKHVVLMCNDPTRSSYKSNLYKPTFAGFVDVDIAKNSKISLRTLIDHSVVESFGENGKTCITSRVYPSLAVGSNVHLFVFNNGAEDIEMSQLKGWEMKNPRMNDGEI*

**Table S2**. The CSP promoter upstream 1k sequence used for this study. Sequence 1k upstream of the promoter of the CSP genes in plants.

> AY493570 Triticum aestivum cold-specific protein gene, promoter region

Acttccgtccatcatgctaggtttagatgccaaaccttgggccgcggtctagacgtcgccgacgtaggacgacggacctttaggcaggggtctacggcttgattctagccggcggcgacgtgcaagacgagcaaaccacgggtttttggccggatccgtggcgggggacgacaacgcggtcagtcgcggcagaggcggcgtcggacatcgggccgttcacgtccgcggtgtcggacggggacggtgagatgcggtgtcgaacgtcgggccgttcacgtccgcgtcgtcggacgggcacggtgagatgcggcgtcgggcggggttgggacggcggcgatcggccagttggaaaaatggaacgggaggagcatgatcgccgggcgggcgagaagatcatgcaactgcctcttttttcccgtacacgggcgatgcctttttttttgcatccgcgcgggtatacgtacgtcggcctgtatgtacaatagaaggtgggtatatcgtttccttcatatggccattctgcccttctacattttgttgggggtctaccgaagcacttctcagaatcctactgtataaaattatttcgaatcaaagccctaagcctctcgtatgcttcttctagttactctcatagtctcattgtcgttacatgccgacactttggatcttccatcctcttaagcaaacaatactaccatttttgcaagagaaaagaaggatcggatatttccggggaatcatcttcttcccggacaaggacgaatgagctgggacgtggcgacccggacgcgccactggcttcagaggcccggcccccctagtcggcagccacctgccgaccactgatgcgaccacacgtagctcccagccgcggcgattcgtccatctgaccagccctctttatgggctagtcggcactcacctgcccatccactcacgagcgcgcacgtcgtggttcgtataccctccaacggcctataaatactgcgtcgcgctgcatatgctttacacaaccacctgcttcacactaccaaggcaagtacacagcagcaatacgtagtagatttcccgagtgaggagctcagcgcaag

> AB676782.1 Triticum aestivum wcr8 gene for cold regulated protein, promoter region

Gaaaaaagtgtcaacaaattgcttcaatgtttttgttatgctttgcttatttagaaagcaaatcatatgctgctctgacctgagattattgcacaaacagattaacctttttttttatcttgaaattgtatctatgcatgtatggaggtgctcctgttgctggaggcagcgctttcagttttcagcggagaccagataattgagatcgattttttccctccttttgggtcatctgttcttacttctgctttggctcactagcattcatctggtgtgaatccccaacctgcattttaatattgcactgaaactatctatagtctagcaaatcatgcagaaattctgaggacattgctctctgcttttgtgttcgcaaatttcctgcttcacagttttgatcccattgcggttgtgattctttactgctaaatcagggggaaacaatttgtggtatgctgcatgtattgacgtggattcagttttctactgtgtataaaaatcactagtatctaagtcattctaaaaatcctactgtataaaaatcgtatgcttcgtctagttactctcatagtcccattgtcttatatgccgacacattcgatcttccaatccccttaagcaaacaatacagtaccatttttgcaagagaaaaagaaggatcggatgttttcctgggaatcatcttcttcccagacgaggacgaatgaggtgggacgtggcgaccgggacgcgcccgctgccttccttatcttttcctgaaagaactatgtggcgctttggctcatcgtatgatgcattcgctgccttaagaggcccggcccccctagtcggcagccatctgccgaccactgatgcggccacacgtagcgcccagccgcggcgattcgtccatctgaccacccctctttatgggctcgtcggcactcacctgcccgtgcactcacgagagcacacgtcgtgcttcgtctaccctccaacggcctataaataccgcctcgtg

> AB297677.1 Triticum aestivum Wdhn13 gene promoter region

Cattaggcgcgtctagttaggggcttagcccaacttatcatattttttattagcagcaaggattatataaacagatataaggcaaccttttggaattaagcaataagtcataactattgcccgactccccttaggagccagaaccctaaaccctagcgcctctcaccaccgccgccgcctcctcccgcgcgatcacggcgccgacgcgccgccagccgcgctcaacctctcaccaacccacctctcatccctacaacctactgttgaggcccggtgaccctagctcctaccagaatgggaccaggattacttatactcaacgaatctgtgaacatagtgctcaactgaacttgtctttgctccggttacagctctcggccgagtgcaccgatctcggagtggatcgagtagccagcgatattgctgtgcttttttgtttaatggaaggttttccaatttcctaaaaaaaaattggaccgtccgctgctggataaatagcaattcaaggtgcgtgcgaaacaatatttagaattacaagacttttaaaccaaagcacaaaatcaagaacatcaagattttttctttagcattcatgcagtctgtaggaattgttctaagatctgtgaaaagtatcccgtgcattaaagaaaatatggtttgcacggtgacggggacgggccctgtgccttggagggtgatatttaaaatccttcattatttgccacacggaccaccacctgtcagcgagacatgttgtggcaaattataaaagtgaaaaaaaaaacaattgggctcctctttattgtcaggtcggcagccactcatgcgagcacacgtcgcgccctgcactgcatgccgcggcgattcgtccatctaaccaccccactttatgagctagtcggcagtcacctgcccacccactcacaagagcacacgtggtgctttctccaccctccaacggcctataaatagcgcctcgtgctgcacttgctttacacag

> AY493570.1 Triticum aestivum cold-specific protein gene, promoter region

Tgcgagacaaaacacgtctggaccatctagcggaccaatataagtccgcgttggatgacttccatgatccagacatatgcgtgatgtttgcgggtcggggatgccctaaacatttcaataccatgaatcaaacagccaacatagaatgatcaaaccccttaacctttcatatgcttcgtctacttactttcgttgtcgtcacatgccgacactttcgatctgctatcttctagtaagcaatacctttttttgcaaacgaaaagaagaaccatcagatgtttttggggaattgtcttcttccctctggtcggcagccacctgccgatccacccatgcgagcacacgtagcgccctgcctcggcgattcttccatctgacccacctgctccaagcaaatgaaactctataaagatattttagtgggtgcacatttttctccaaaaagtaaagtgcaaatgaattctaaggaaaattaccaagcatatcccctaaaaaaagtacctagcatatcagtcctatgaatgcaatagccaacataggaaaatacctaggatctagatcgtctaaaatcctataaaactcttttgaatcaatgcccttaccctctcatatgcttcatctgtatattttccttgtcgttgcatgccgacactttcaatctagtatcatcttcttaggcagacaataccattttttgctagagaaaaagaaggatcagatggttttggggaactcatcttcttcccggatgaggatgaatgaggttgggacgtggcgaccgggacgcgcccactgccttgccttcagaggcccgggctcctctagtcggcagccacctgccgaccactgatgcgaccacacgtagcgccctgccgcggcgattcgtccatctgatcacccctctttatgggctagtcggcactcacctgcccatccactcacgagagcacatctggagcttcgtccaccctcgaacggcctataaatagtgcctcatgct

>AY493570.1 Triticum aestivum cold-specific protein gene, promoter region

Tcgcaaattgcctgcttcacggttttgacttcattgtggttgtgtttctttactgctaaaatacgaaggattgtagtgacatgtcctgaatccattgaattagaaaactacatgaattcgaataaaagattgtttgatattatccctataaaagcaaaggaagtctacaaagatatttcagtggatgcgcatttttctccagaataaagtgcaaatgaattttaaggaaaattactaaacatttcaatcctataaatcaaacagcaacatagagtgtacatttggcttcaaaatttgtccacaaaagagtgtacttctatcttctcaatgcactttaaagtagaaaaacatacttctctctcatcacatggtaatcaagacaagtaacaatctacacatgatctttttaatttctacatacacttagctcattagggttggataattaaagatgagagagatgatggcttgcatcttttcaatacattttttacttcacttcataatttgttctaaaatctctaaggacaaattaccggacggagagagtaaatcgtctaaaaatcctataataatttcttttgaatcaaagcccttagcttctcgtatgcttcgtcgttacatgccgacactttggatcttccatcctcttatcttaagcaaacaataccatttttgcaagcgaaaagaaggatcagatttttcggggaatcatcttcttcccggccggacgaggacgaatgagctgggacgtggcgaccgggacgcgccaactagcttaagaggcccggcccctctagtcggcagccatctgccgaccaccggtgccaccgcacgtagcgcccagccgcggcgattcgtccatctgaccacccctctttatgggctagtcggcactcacctgcccatccactcacgagagcacacctggagcttcgtccaccctcgaacggcctataaatagtgcctcgtgctgcacctgctttacacaaccacttgc

>NC_003071.7 COR15A cold-regulated 15a [ *Arabidopsis thaliana* (thale cress) ]

GAGTATTAGGTTCTTAGAAATATATCTATATACTATTAGTAGTTTAACTACAGTTTGTACTTAATTGAAAAAATGTTAAAAGTTGTTTTAACCTAGCTAATTGCTAAAAATGACTAAATAGACATACACAAAGACTTGTACATTTTCAGCTTAACGACTAATACATTTTTCCTTTATATATATATCTCTATCGAGTCTAGTTATTAATGTTGAAAGTTGCAAATAAAACAGAAATGCTAACATGTAAATATCGTAGCCAAAAATGCTAACATGTGTATAACGGTTATAACCACAACTTGATGGCCGACCTCTTTTTTCTTTTGGTAACCATAGAAATGGTTACACGTAACTAGTACGAACCAACGAAAACTCTTCTTATTCGATAGTTAAAGATAATAGCAATGCGCAAAAATATCTAGCACTCACACGTGTAGTTTTGGATTCTCATTGGTCGAGAGATCTATAAAACGATACTATTGGAGGTTAGATTTTTCTCATCTCACTTTCTCCATCTTAAAACTCTTTCTTGTATTTATTTTCCTCCCAAAAAACATCTTTAAGAGTCCTCATGGCGATGTCTTTATCAGGAGCTGTTCTCAGTGGCATGGGTTCTTCTTTCCACAACGTAGGAGCAAAGCAGAGTGGTGTTGGTACCGTCAGAGTTGGCCGGAAGAGTGAGCTCGTCGTCGTTGCTCAGCGCAAGAAGTCGTTGATATACGCCGTTAAAAGTGACGGCAACATCCTCGATGACCTCAACGAAGCCACGTGAGTCTATATTCTTTTCTTTTAACAAATTTTCTCGTGACTAAGGCTATCTTCATGTGTACTCGCAAAAACTCTCATATACATTACGTACTCGCCATGATAGTTATTTTATTTTGGTTAATCCCATCATAACAGTTTTATACATACTATAATCAACTATAATGGCCTTGTTGAGGATTGAGTTTCCATGGTGTAGATATATGTTTTTAAAATCGTTTGTCAATAATGAATCTTAACATATATATATATATATATAGAAAGAAAGCTTCTGATTTCGTGACGGATAAGACGAAGGAGGCCTTGGCGGATGGCGAGAAAACAAAAGACTACATTGTTGAGAAAACCATTGAAGCCAATGAAACTGCGACTGAGGAAGCTAAGAAAGCTTTGGATTATGTGACTGAGAAAGGAAAAGAAGCCGGAAACAAGGCGGCTGAGTTCGTAGAGGGTAAAGCAGAAGAGGCTAAGAATGCCACAAAGTCCTGATTCAACCACTCATGTAGTCATGTTCAATAGTTTACAATAAGATCAGTTTGTTGTAATTTCTTTTTCACCATTTGAAATGAAGAGCTGTTAATTACTCACTAGCACATTTTAAATACGTATCAAATGATTTAAATATAAACTACATTTTAGTGACTACAAGTAACATAGTAGTATTTATGAGTTGGATTCGGGAAAAATCTGTCAAAATCTTCTCTCATTTTGATCCTCAATTATAAAGAAACTGTGAAAC

> NC_003071.7COR15B cold regulated 15b [ Arabidopsis thaliana (thale cress)

CTAATACTCCAATGTTAAATAAACAAAAACAAAAACTCACACTTCAATGCCTAATTGTTATACTATAATTATAAATACCAATGTTACTTTCAAATATAACTATTTTGCTCAACAGCAAAAACGCTATTTACAATGCCTAATATTTTTTTTTTTGATAAATATGAAATAGAGCATGTACCAGCCAAGAATGAGCTTATTAGATATGTCCCACATTGGTTGGGTATGTGAGTAATGTGCTCCTTAATTATGTGGTGTGGCATACCGACTTCTAGATGATATGTTTGTGACGGGAGATGAGGTGATAATTTGTTGGTGCCGGACCATAATTTTTCCTTCCACGGATTGTAATTCACTGCTTGGAATGGCTAAGTGGATCAATGTTGATTACAAGCCACCAAATTTTGAAGTTTTTGGTACAACAATGTATCATACCAATTCGATGTCATACCAAACAGTAAATAGTGGACATGCGAAGAAAAAAAGCAGTTCCTCTTTTATTGGTTCGTCACAAAAGAGACAAGGCTATCGATCTCCCATATGCTCTCATCCTTTCACCGTTGAAAGACTCTAGACGGCGATGTTACTTATAATTACTCATTATTTAATTTTGTCTATTGAATATTTTGCAAAACATTAAAGAATGTGACGGTGACTGTGGATACCATATCTTATTTCAGTTGTGATGTGAAATTAATAGAAATTACAACAGACTCATCTTATTGTAAGTACTATTAAACATGAAGAGAGAGGATATGGATCAATATATCTACGAGTTTAAGTGCTTGAAATTAACTGATTAGGTAAGACCCTACTTTGTGGCATCCTTAGCCTCTCCTGCTTTACCCTCCGCGAACTCTGCCGCCTTGTTTGCGGCTTCTTTTCCTTTCTCCTCCACATACGCCGCAGCTTTCTCAGCTTCTTTACCCAATGTATCTGCGGTTTCACTGTTTTTTTCAACAACGTAGTCTTTCGCTTTCTCACCATCTGCTAATGCCTCTTTTGTTTTATCCGTCACGAAATCTGAAGCTTTCTTTCTATATCATTTTCAAATTAAAATTTATTTTAGTATTTATCAAAGGTTTCAAAACACATATCCAAAAGTAGTCAACAGAATCATCAAATAAACTTAGAGCTATACATGTAAACTAGAAAAATAGAGATATATAATGTACGGTATATGAATCTTTATCTTTGATAATAGTCAAATATGATCATAAAATAAGTTCAAAACAAACATATTTCCACTAAAATCTATACTAATATAGTATACCATAAACATTGCCTGCTAAGATCCTTACTTATGAGGCAAGATACTAAAAGAAGAAAGAATGTAGACTTACGTGGCCTCGTTGAGGTCATCGAGGATGTTGCCGTCACCTTTAGCGGCGTAGATCAACGACTTCTTGCGTTGAGAAACGACGACGAACTGAGTTTTCTGGCCGACTCTGACAGCGCCGAAGCTGCTCTGCTTGGCTCCGCTGTGGAAAGAAGAAGCCATAC

>NC_003076.8LTI78 low-temperature-responsive protein 78 (LTI78) / desiccation-responsive protein 29A (RD29A) [ Arabidopsis thaliana (thale cress) ]

ACCGTTGGTAAAACTCCCTCGTCCTTGTGTTACACGTAATGTTAAGATTTTGATGTGTGTATTATTTTTGTAATTTTACTCAAAATGTTTTGGTTGCTATGGTAGGGACTATGGGGTTTTCGGATTCCGGTGGAAGTGAGTTGGGAGGCAGTGGCGGAGGTAAGGGAGTTCAAGATTCTGGGAACTGAAGATTTGGGGTTTTGCTTTTGAATGTTTGTGTTTTTGTATGATGCCTCTGTTTGTGAACTTTGATGTATTTTATCTTTGTGTGAAAAAGAGATTGGGTTAATAAAATATTTGCTTTTTTGGATAAGAAACTCTTTTAGCGGCCCATTAATAAAGGTTACAAATGCAAAATCATGTTAGCGTCAGATATTTAATTATTCGAAGATGATTGTGATAGATTTAAAATTATCCTAGTCAAAAAGAAAGAGTAGGTTGAGCAGAAACAGTGACATCTGTTGTTTGTACCATACAAATTAGTTTAGATTATTGGTTAACATGTTAAATGGCTATGCATGTGACATTTAGACCTTATCGGAATTAATTTGTAGAATTATTAATTAAGATGTTGATTAGTTCAAACAAAAATTTTATATTAAAAAATGTAAACGAATATTTTGTATGTTCAGTGAAAGTAAAACAAATTAAATTAACAAGAAACTTATAGAAGAAAATTTTTACTATTTAAGAGAAAGAAAAAAATCTATCATTTAATCTGAGTCCTAAAAACTGTTATACTTAACAGTTAACGCATGATTTGATGGAGGAGCCATAGATGCAATTCAATCAAACTGAAATTTCTGCAAGAATCTCAAACACGGAGATCTCAAAGTTTGAAAGAAAATTTATTTCTTCGACTCAAAACAAACTTACGAAATTTAGGTAGAACTTATATACATTATATTGTAATTTTTTGTAACAAAATGTTTTTATTATTATTATAGAATTTTACTGGTTAAATTAAAAATGAATAGAAAAGGTGAATTAAGAGGAGAGAGGAGGTAAACATTTTCTTCTATTTTTTCATATTTTCAGGATAAATTATTGTAAAAGTTTACAAGATTTCCATTTGACTAGTGTAAATGAGGAATATTCTCTAGTAAGATCATTATTTCATCTACTTCTTTTATCTTCTACCAGTAGAGGAATAAACAATATTTAGCTCCTTTGTAAATACAAATTAATTTTCGTTCTTGACATCATTCAATTTTAATTTTACGTATAAAATAAAAGATCATACCTATTAGAACGATTAAGGAGAAATACAATTCGAATGAGAAGGATGTGCCGTTTGTTATAATAAACAGCCACACGACGTAAACGTAAAATGACCACATGATGGGCCAATAGACATGGACCGACTACTAATAATAGTAAGTTACATTTTAGGATGGAATAAATATCATACCGACATCAGTTTGAAAGAAAAGGGAAAAAAAGAAAAAATAAATAAAAGATATACTACCGACATGAGTTCCAAAAAGCAAAAAAAAAGATC

>NC_003076.8KIN1 stress-responsive protein (KIN1) / stress-induced protein (KIN1) [ Arabidopsis thaliana (thale cress)

CCCAGTCTTCGTAGAGAATCGCTACGTTTTTATTCTAACCGAAACAAGACAAGAAACGTGTTTTCGATTGTGTTTGTGTATGCCGATAAACTATTATATTTTTGTAATTTGATTTAGTTCAAGAAATTGTTGTGGCCTATCGGCTATCAACATGCTTTTTGTAAGAGAGAACTTGTAATTTCTGTCTATGATTTCCTTCGTTTTCTTTTTCTCTCTCAAAGTCATTTATTTAAAGAACAACATTACGTGTGTATAATTGTATAATTTTTTCTATTTCTTACTATACTTAGAGAATTACATGTTATAACAAATGATCTTTGATCAAAAGAAAAACAACAACAAATGATCAAGAATTGCTTTCTTTTTTTTATATGTTGCAAAATGAATAGACGAGCCAAACTTATACTCAAATATTGTTTTATCTATTTTTAAGAAATATTCTTTATGCGAGAGAGCAAACTCCCCAAACGATGTTAAATGGATCACAACACATAAGTCGATCCGAATAGTTGAAGTTTTCTAAAAAGGCAGTCCCTATAATTGTATTAAACACACTAAATCCTCCACACAGACAAAAAGAAATCACGTAAACGAGACATTTACATGAAACAATCAAACCAGTTTGATTTTATCCGATTTTGCTAATGAGATGAGTTCTGTATATTGTTATAATGGATATAGGAGAACTAACTAAAATAAAAAACGCAAAGAAAACTTTATACAGTACATATTCTTACGGTCTTACCAATTATCAACGATGTGTTGGTTATGTACACAAGGTAAAATACATCGATTCATATTATGCATAATATAAAAAAAATAAAGAATAAAACTACATTTTGGTAACTTGAATTCAACCATGAACTGTTTGGATTGGCAAACATAAACTCAAATAAAATATCTAGGTATAATTGTGGTTCATACAAGAATTACTTCATACTGTTGGGCCAAAGGACGTATCCTTCCCCGCACCTCCAAACCATGGGCTTACTACTGATCCGACATCAAAACCGTGTTAGTTGCAACCAACGAATGATAAGTCAATAAGATTCAACTTGTCAACAAATATACAGCTTATATGACATGTCTGGCTCCAAACTGAATTTTAGTAGAAAGTTACTAATTCATAAAATTAATTTATATACAATTTTTCAATTTTTATTTTATAAATTAAAGAAAAAAACATGAAAAATACGGGAGGTTCGGCAAACACAACATTTAACTTGCCAAACGTATCATCTAACTTTCCCACCTTATACAAGGAACCATTTTTTCAATAATAAAGTTTTTTTTTTTTTGTCTTCGCAAATAAGAGCACGAAATGTTTGCCAAACGCATATGCAACAAACCCACGTTACATAATTCTGTTTACAGCCATAGAGCAAGCTATATTGTTAAAGACCTAAAAAAAACTTTACTATAACATATAGAGGCTTCGAGATATTTCGAAAGACTCAACTTATATATAAATAAACTCAAAAAGAAAACACGGAGGCGAGAG

 >NC_003070.9ERD10 Dehydrin family protein [ Arabidopsis thaliana (thale cress)

GTCATGGCCTCATGGGGATAGTAAGATTACTGTATGAACCTCTCTTTTTTTTAATGGATTTCAGTTAACCAAATTATATGCTAAATTTAAATGTTTTTACCATTTAAAATATATAGATTTCAGTGAATAGAGCTATAATATTTTATAGTATGAAAATTCTTTTACCATTTGAAATATATAGATTTCAGTGAATATAGCTATATTCATAAGTCATAACAATAGACCGGTTCAATATCACATTTATGAATCTATAGTATATTTGTTCATAAACGAAAGTAGTGATTACTTCTTCATAGTAGTGAACTAGGCAATGGACTTTTCAATTATTAAAAGAAAGAGTTTCGATAGTCTAATTGGTAAATTCTTGGCACATAACATTATCATATTTTGGGGTCAATTTTATAGATTTTCAGTTTAAAATAAATAGAAACCGGTTTGATAATGATTTAGAACTTTAGATTCAGTTTAATATTGTTTTTAGTCATATACAGTAACATTTGACATTTCTATAAGAAAACTAGATTTTTTCGTTTTTGTTTTTTCCTTTTAAGCTCAGGTAAGTTTACATGTGGTGCACAAAAATATTTGTTGTAAAGTTTAACGTAACAGATTAATTAAATCAATGTAGCAAACGAAAACGATTGCTTTGTTTGGTATCATGTGTCGGTAGAGTGACCACCCCAATGCCTAAACTTTTTAAAATTCGGTCTGTTAATTACGGGTTGCGTGTTCATGAAACGTCTCAATTTATCAAGTGGCACAACGTCTTAAATCTTTCAACCGAGCACGATTCATATAATTTAATGAATTTAAGCAAAATCCGAAATTGTGCGTTCTCAAATGCCCCATCTGTTAAATTTTATAAGGAAAAAGCTTTCGTCTGTTATCATACAAGGCACAAAACGACATCCCTTACCTAAACTGTTATACTTATAAAAACAAATTGATTTCAAAATTAAAATCTGCTATGCTGCATGTACATTCTTTTGGTAACGTCTTAATTCTTAAAAAGGATTAGAGGAGTGACAAAAAAAAAAAAAAAAAAAAAAAGGATTAGAGGAGTAATGGCATGTTAGTAATTTACCCGAAAAATATCTGCAATCTAGCTTTAGCCGACGTGTCTAATGGCCCACATGACCGACATCTATGCTTAAAGCCAATCAAAGCCGACCATTCAGCTCCATGTGTGGGCCCGTTCCCACCTCTTTTAATACGTGTCAGTAAATTATTGGTTCAATTCATGCCTTCTACATTATTCTGTTTTTCTTCTAATTCGTCCTAACTTCTATTGTATAATTAGTCTTTCCATTTAGTACTGATCCTTTGGCTAGATAATTCTGTTAATACTTAATAGTTCGGTTATAATTCTTCCCTTTTAATTTGCCCCTTTATTTATAGTATATTTTACAGATACAACCTGAAAGTCTTAAAAGAGTTGGAAACATCACTTTTGAAGTTTGAAATTTCTTTCATATGCTTTTTATATAATATTTTTCAACAA

> NC_090805.1WRKY transcription factor 31 [ *Solanum lycopersicum* (tomato) ]

GCGGCCAGTAAACTGATCAAATCTTTTATTTTGGCCACAATCATTTAAATATATAATTGCTGAAAGAAGAATATACACCTCTAGAAAGAAACTTTTTCCTGTTCCAATCGTTATCCATTTTAAGTGTATAAACAATTAAATGAATAAAATAACGGGAAATATCCATTAGAGAGCATGACATTTGTTATTTGTCCAATAAAATTTGATGTGCTTGCTTCTTTGACTTGTATACATTTAGCTTAAATTGACTTTATACTTAAACAAATGAATTTCTCTTTAATTAATAATCAGAGTAACGTAATCCTAAAATTCTAGTATATTAATCATAAAACATATAATAAAAACACATTGTAAAATAGCACTTGCAACATATCTCGACTATCTAGACTGATATCGATAATATATTCTGAGAAACTTCTTTTGTGATCATTTATTACTTATTAGGTTAAAGATAAAAACTCCTTTGTTTTGATGCAAATAGAATAATACACAACCACCTTTAACTCTCACTAATGAGCATAAATGATTAAAATGTTTAAAACAAAATTGAATTGTATCATTAGAAAATTAAAAAAAGAAAAAAGTTAATTCATTTTTTTATTGCTATAAATATTTAGTTTTAAATCTATCCAAACAAGGTTCGAAAAGAATGGAAAAAGATTAGATAAAAAGGATATTGACAATTCGCCCGATAAAAGAGCCTCGTGATTGATTGATTGTTAGTTAATCAACTGATAGTGGCCAGTGGGGTACCACCATCACAACGACAGCGACATAACGCGTTTGGCGTAATTGCATTACAATAGCAGCCTCTCTTTTCTTGTACAATTTGTGTAAATGTATCTATTATAGTCAAAGCCACCAATTTTGACTCAACACACAAATAGTATTATTTTTTAGATTTCATTTTCCACGGTCAAAGCTATTTTGAGGATCTTAGAAGTTTCTTTAAATATCTATAGAATAGAAAAATAAAAAAGACAATGAGCTTGGATGACACTGTTTTATTTTTTAATTAAAAACTTTAGAATTTATATTTGACAGAAAGGAAATTTTATTAAAAATGTCAATTATCAAATAAATTATTAAATTTAATCAAATTTTAATATTATCGAGTGAGAAATGAAAAAAAAAAAGCTAGTGGATTGTAGAATACGTGTTCATTTTTGTTTTTAATATCCCACCCACTCCAAACGAAAGGTTATATAATCGAATTCCCATTGCACTCTTGTATCTTCTTTCTTTTAATGGCTGCTTCAAGTTTCTCTTTTCCCACTTCATCTTCTTCATTCATGACGACTTCTTTCACCGACCTTCTTGCTTCTGATGA

> NC_090801.1WRKYIId-4 WRKY transcription factor IId-4 [ Solanum lycopersicum (tomato) ]

TTGAAAAATATACTTAAGTTTCTGATGTTTACGTGACATTTTTAACATATAAAATTAATAAAGAAAATGATGGATTAATAACTGTGCAGAGGATAATTTACTCAACTATTTAATTATTACTGAGAACGATCGAATGCAAAAAATAATTTACAGATTCATTCTTTAGTTCATCAAAAAAATAAAATATAATTAATAAATTTTAAACCCAATTACTTACTATAAATTTTTTCCGACAAGGTAGTGCTATACTAGAAGTTCTTTTTTTCTCATTACTATTCTTTTTGTTTAAAAAAGAATATTTTTTTTCCTTTTTAGTTTATTTAAAAAAGAATGACTCATTTTCTTTTTTGGCAACACTTTAATTTTTAACTTTCCAAATCACATGTTAAATACCACAAGATTAAAGTTCATTTTGATACATTTGACATAACTTTAATTTAGAACCATGACATTAAAAAATCTTCTTTTTTAAAAAAAAAATAACTTCGCTTCGAATCAAACTATGTTACCTTTTTTTAAACGAATGAAGTATTTATTTAGGAAATTACTTTTACTGACCTATGATTCAATGGACAACTCACCTAAAGAGATACTGATGAGTTTTCACAAAATTTGCTCAAGTACGGTACTTGTCGTGTGAAAGGTTCGTTCAACAAACATGTTTGACTCCAAATTCATTAAATTACTAGTACTATCCTAACTGATTTTGTGCTTAATTAACCCATTTAAACAAACTTAATTAAACCATTTAGCCCTACAGTAAGAAAAGATTAACACTGAATAATTACGTATAAAACTTATACATCTTAAGACGTTTTCTTGGTAATCCAACAAAATTATATTTACCATAGTAAAATAATAATAACAAATTATAAGCTTTAATAGAATGGAGAAATTCCTTTTTACATACAATGAAAAATAATATATATATATATATATATGAAAGAGTTTTGATATTCAAAAAGATAATACAAACGAAATGACTAAATTTTTATTTTAACGAAAAATGGATTCAAATTGAAGAAAGAAACGAAATTCATTTAGCGTTGCATGGTTTTTATTCAATTTGAACGAGTCGTAAGCTTAAGTCTTTTGTTTTTATTTTTTTTCTACCATTTGGAAGAGAGTTTAGTTCCAACTTACAAGAATGATAAAATTCTCCTAGTTGTTTTTCTTTTCTAATTTAAACAACTTTTTTTTTTTTTGTAATAATTAATATAAGTTAGTCAAGCTTAAAATATATTGTCAACGGAATTATGCTGGCTAAAACCAATTGAGGGGGTAAGACTAGTAAAATACTTTATACATTCTAGAAGAAGATGAACCCAACCATCAAAGATAAAAAATCAAACTTAAATAATTAAAGTGGCCAAAATGGAATTGAAACGAAATTCCTTTTATTCAAAAAAGAGATGAGGACAAATGGAATTGAAACAAAATTCCAACCCTGCTAAGTAAATTAAATGATATAATACAAAGACAAAAAGATTCAGAAAAGTCAACTAAAATGGGAGGGGAAAAAATGAAGTAGAAGAAGTAATACGTAACCGAAAACCACTAATTTATATGCGTTTTACATCTAAATCTTACTTATAACTTATAATTTAGTGATAAGTTATGTTTTTTTTTAAAAAAAATAAATAAGTAAGGACATATCAATGCTATTACAAAGTAATGAAAGATCTTGAATAATTTTTTCCTAGATCCATAACAATTGTATTTTTCTTTGATGTTCTAGTATGTATGTTAATAATAATTAAAAAAAAAATGGTTACTAAGAACAAATCCTCCAAATCAAACACAAGCATCTCGTGAATCAGACGGCGTTGTCACACTCCTTTGACCTGATCAGTTCAAATTTCCCCAAAAGAGACCCCACTCACTCTTATTTCCCCAAAGAAAAGGTCAATGAACCATTACTCTCATGACCCACCCTACACCACAACAATCTAAATCATTTTCCCCTTTTATTAAAG

> NC_090802.1WRKY72a WRKY transcription factor 72a [ Solanum lycopersicum (tomato) ]

CATGTATGATTAGTTTCTATGTAATTTGTGTGACTTGTTAAATTTTATAACACTAGTTTTAGTTATAAATAGCTTGTAGGAGGGTAATTAGTTAAAGGGTAATTAATTATATCCAGCCAGATCTCTTTAAAATATAACTATTAGTGCAAAATCTTGATATGATATGGAAGCAGATCAGGAAGATGATTTAGAGTACAAACATATGATGAGTTCGTTAAAACCTAATATACTAATAAATTCACGTATTTGATTGTGAACTGCTTTATATTTTATTGTACTCTCTCCGTCCAAAATTATTTGTCATGGTTTCTATTTTTAGAGTCAAACTATAAAAAAATTGACTAACATTTTAAGATGTATTTTCTCATCATATTAATATGCAAAAAATTATAATTTATAGTACTTTTCATGTAGTTTTAGAATATTTATTTTTTTGTTTAAAATATCAAATTAATGTGATCTAATTTACCTTTAAAAATTAGTCAAATTAACTTACAAATAATTCTGAATGGAGAAAGTACTGACTTAATTGAAAGATCATGAAAAAAATGAATATTAACTTGAGACAGCTAGCTAATTATAAAAACTCATAAATATCCAATTTTAAATCTACTTCTTAGGAATTATAGAAGGTTGATGTCTTCCGCATCAAGGCACATTCATATATACTATTCCCTCTGTCCAATAATAGTCGTCCACTATACTATTTTAGAATATTCAGACTGGTCTATATTATGAAATCAATGGATTATTGTGCACTTAGCTTTTAATTTATTCTTATCATTAATTATAGTTATTTTTCTATTACATTTATCAGGATATTATATTTAAAAAGTGATAATAACAAAATTATATTTTTATTTATAATTTCTTAAAGAAACCTCTAAAATCGATTGGAGTATGGTAAATAGTCCTGAGAACGTTGAAAGGATAGAATGTGGGGAGTTGATAAAAATGTGAAATCGTAAGGTAATTGGAGGATCCAAATCACATGCAGGATAAGTCAAACCAAAAATATTTTTTGTCAACAACATTTAAAGTCATACTAACCGCGGCAGAGTTGTCAAATATTAATTTTCAGTCTTCAATTTCAAAATCTTTGACTGTACCAATTAAGTAGCTAGGGCATAGTCATCCTTTACTCATGATCACAACCGACCTCTTTTGACTTTTGGGTATAACCTACTTTATTTGATAAATCTATTAAGAAAATAATAATTGATATAACGAATAAATTTATTGATATTTTTATTTATTAATAGGATCTCAAACATATTTATTCACAGACATCCTTTACTCATGATAGTTTTTAAAGATATTAATTCACTGTTAACAAAAATTGAGGGTATATGATAAAAAAAATTATCCTTAATTTGTCAAAAATGACAAGTAAAAGGTGAAATTAAAATAGAAAATATTTGGCAAGGGAATAGAAACATTGGAAGTACTTTTGTTGTATATATAATTGTTTTCCCTTATAATTTGCCACGTGATTATTTTATCACTATGTTAAGACTAATCAACACACACAAAAAAAAAACTTAATTATCCTAATGGTTCATTTTGCAAAGATGGTGTGTTAAATAGAAAAGAAAAAATAATACAAGAATTTAACGAAGTTCAACCAAGTAAGTACATGTTTTCTTTATTCATTGTTGAGAACTTAAGTTGCTATTACTG

> NC_058151.1dehydration-responsive element-binding protein 1A-like [ Mangifera indica (mango) ]

TCCAAATTTATGCATTCCAACCATCTTCGACCCTAATGGGGAGAGGATTCCCCAAATAAAATAGGGATAGAGAGGAAAAAGATCCTCGTAATCAAGGATGGGGATACATATGTCCCCTCTCCTCACTTCGCCCTAATCCTTTTATGTTAATATATTTACTTAAAATACTAATATATTTTTATAGTTTTAGATTATTTATATTTTTCAAATTTATTTATGTTTTCATTGTGCATATTAAGTAGTTAATATATGATATTTGTGATATTTGGAATAGTTGATTGATTTTAATTTTACTTAATTTTATTATTGAATATATTTATATTGTGTTTAGAAGATATAGAAATTAGATTTTTTCTCATGGGTATAGGAAGATGATTTTTTTCCTCGGGTACAAGGAGGGGATAAGGGATTTTTCTTCATGAGAACAAAATGAGGAATCTTTGTTATCTCCTTCTTATTGCTATCTCTAGGGAGAATAGTTATTTTTTAAACTTTGAATAAGAGAAGAGTATTAACTTTTTAAACTAAAAGAAGATAGATGTGATAAAATTTTAATTTTTTAATATTTTTAGTTAATGATAATTTTATTTGTAACCCTAACAGAATGATAGGGTTTTTAAAGCTTTATGAGTGGGAATTTGAAATTTGACAAAATCTTGGGTGGTAAAAATTTTTGGCCAAAAAAAAAAACCTAAAACCCACTTTCCTGCTTGTTGGCTTGCTTGGATTCCACAAAAATATATTTGGGTAGAAAAAGAATAAAGAAAAGGCCAAAGGACTATTTCCCGCCTAAAGTATGTTGTAATTTTAACTTTTCATCTGTTAACTATGAAAATCCTATTTACTCACCTATGGACTGTTAAAATTAACAGTTTAAAAGGTAAAATCGTCATTTTATCTATAATATTAAAAATAAATTAAAATTTAATCTAGTTTTTCTCCCTAACCCTAAAAACTAACCATTTCCCCCTAGGCCAAATTTTAAAAAATAGCATTCCCCCTAGGGTTTAGCTTTCAATCCCTAACGTCAAATCTGGTGTCATTGCCAATGACAAAGCTTTCTAGAGGCATCACCATGCTCCGACCATCTCTCTTTTCTCCTCTGAAACGTCAGTCAAGAGATCTGACTTGGAAAGAGGGATATCTTCCTAGACGAAGACGACGACCTCATCTTCGTTTGGGATGTCTTCCCAAAAGAAGACGTCTAGGATCTCGATCGATCGATGTTTCAAAAGAGAAAAGAGAGACGGTCAGAGCATGATGATGCCTCTGGAAAGCTTCGTCGTTGGCGATGACGTCTGATTTGGTGTCGGGGATTGAAAGCTAAACCTTAAAGGGGAATACTATTTTTTAAAACTTGACCTAAGAAGAAATGATTAGTTTTTAGGGGTTAGAGGGTGAAAAGAGATAAAATTTTAAGGGCTTAGGATTCTGTTAATTTTAACCGTTTATGGGTGGGTAAATAAAATTTTCAAAATTAATGAGAGAATTTTGAGATAATAGCATATATTAGGTGGAAAATAGTCTTGTGGCCTAAAGAAAAATGAAAAAATGAAGAAGTCTCGACTCCCCAAACACGGTTGATGATGTTGACGCTCTTGCTTTCCTTCTGACCAACTTCCTTCGGTTACTTCCAACTGTCGATTCATTAACTGAAAAATAAAGAAATAAAAACAAGGGAAAATCAAAATCTTTTTAAAAATGGTGAACTCCAGTTAAGCCACGTGGTCCACACGAAACCGCGGAAAAACCAACCCCCCTTCCCTATAGATCCGTTGGGCTCGCTGGGTTTTAACTGTCAACCCGTGTTTTGAAATTCGTGTGACGTCATTTTGAAAAGGCTTTCCTTTGTTTTATATGTGCCACGTCATCACTTCGTTCAAACCACCACGTGTCAATC

> NC_090800.1GRAS4 GRAS4 [ Solanum lycopersicum (tomato) ]

TTAAAAATTTACAGTCACAAAAATATTAATAAAAAGTTTTATTCGTTCCGCTCACTTTTAATTGTTATAGTATCCTTTTTAGAGTTAAATTAACATTTTACGAGAATGTTTTTTATCATATTAATATGCTAGAAATATAATTTATATATTTTTCATATAATTTTTTAATATATAAATTTTTTACTTAAATATCAAATTAACGTAATTTAATTTAATATTACACAATTAGTTAAATTTGTACCCATAAAGGATCATTTGCAGTATATTATTCGGTCCGTTTAATAAAAACAGCAAACTTTAATATCAGTTTTTCGATCGTAAAATTTAAAAATATTTTGAATATAATTTAAAATTAGAAGGTAGAACCATAAAATTGAAAATCTTTTTTAATTTAAGTGTTAAATTAAATTAGATCTTCTGTGAAACGGACGGAGTATATGCTATATATCACAAAGTCAACTGCACCTGCACGCGTGAAGATGTTTCCAAACAGAGCAAAGGCCGTAGTCAGGAGAAAGGATTTCCTGTTATGGGAGTGAAAGTTATATTGCTGGTAATTCACTTTACAATTCAATTTTTCGTCAAATTAATTGGTAATTCCATCCTGTTGTTTCTTAGAAGAAATGAATTCAGTTACATGATTTACAGGTGATAGTATAATAATAAGAAACTTGTTCTGGATTATATGTCTATATGCATATATAATTATTATTCACTATGTTGATGCCGTTGTGGTGAAGGAGACAGCATTAGCTTGATAGTGATCTGTGTCGTATATAGTCAAATTCTGATTTGTCTAATCCAAAATCCAAATGCACGTAAGCTATTTGAGTTTAAGCTTCAAAGAAGGCTTTCATTTGTAATGCTTCTGCACGTCAAGTGAGAGAAATTTTTGAAGCATTTGAGAGAGTGAAACTAAATTAGAATAAATTCCAAAATGCTGCTGCTACAAACAAAGATAAAACTCCACTAGCATAGGAAAATACAGCCATAGAAGAAATGGCCTGATAAACTAGACTAATTTTACAGACTGCTTTGTAGGCTTCCAACAAGAGAGAGCGTAAATTACGCGTCCTTTCCATCCTTGTAGCATCCAGTGTCCATCTTCATCAACTGAGAAGTCCCTGTGGTACTCCGACCTCACTTTATTCCTCACTATCTTCACAATCTCCTGGTCAAGTGGCACCTGTTTGAATCCAGCTCTAACGCATCTAAGTTGCCACTGCTTGTATGTTTCAGGTCTCTCAACTCTCTCTGTCCCTTCACAAGCAATCACGTTCATAGCATCTCTTGCAAAAACCTCTTCCTCGAATAGCTTTCTGTCCTCGTCCTCGCGGGGAAGAGTAGCCTCAAACATATCATACAGTGAAGAGAAGTGAAAAAGCGCCTCCCTGAATCGTGTGACGAAAAATGGAGTGTTGAAA

> NC_088327.1WRKY transcription factor 3 [ Musa acuminata AAA Group (dessert banana)]

TGCTCTCCGACCCTTTCTGCAACTCCTCAGTTCTCTTTGTCGTCTTAAAGCTCTGAGGCTGCTGTCTCTTAAGCTCTCCGAGGAAAAGGTCTGAGAACCGCTTTGCAGATCACTCTTGTTTTATCTGCTTGACGTTCCATTCTCCTTCCTTTCTTGCTGGAGCAACATAAGTTAGATATGTATCTGTTCCTTCTGATGTTGGCTTTCCCATACATGCAGCAGTACGCCATCAGCTCTCGCGTTGCGGAAGTCTGTTGGTGGACGGATTCCACTGGCTTACCGAAGCTAAATGGTGTTCGGCGTTGTCGATTAGTTGATCGGCGGAAGATGCAAGATGCAAAGAAGATTGAGATTGCCAAACCTGTGGCGTCGAGACCTTTCGCCAGTTTCAGGCCACTTCCTGAGCTCCTCGCGAGAGCGACCAGTTCCTCTCCGACGCCTTCTTCTGCTGAGAGAACAGTCGCCATCAAGCCGAGGACTATGAGATTCAAATCCTCGTCGTACGATTCTGCAGCTGAAGCTGTTCCACCGCCTGTGAGTTCAATCAAGATTATGTAGTTTCCATGGTTTCTGTGTGCATGCCTTTTCTCCCTCTGTGATCAGTGAAATCTCCTGCAGGATGATGCATCCGAGAAGAGATCCGTGGCAACCATGGGATCGGATTCCGTGTCAAGCTTCATCTACAAACCTACTGCAAAGCTTGTTTCAAGAACCCTTAATCTGGTCAGCGTGCTTTTCCTTGCCTATGAGTCAAATGGTGCGATCTCCTTAGATATTAACTCCATTCTGCTTATTTCGAACAGGGAAACTTCGATCAAGTTCTTGACCAAGTAAAAACATCAAACAGCCGACCTCAGAATTCAGCATCAGCTTCACAATCTGAACCGAACCAGGCATATGATCCTTCCAAGTCAGTGACGGCCGGCGAGCTTGATACGAGGAACCAGCAATCGGCAGATCGGCCGTCTTATGACGGCTACAACTGGAGAAAATATGGACAGAAGCAAGTAAAAGGAAGTGAGTACCCGAGAAGCTACTACAAGTGCACACATCCCACTTGCCCTGTAAAGAAGAAGGTGGAGAGATCACTTGACGGGCAGATAGCTGAGATCGTGTACAAGGGCGAGCACAACCACCCCAAGCCTCAGCCTCCCAAGCGGCCCTCGTCAGGTTCACAGGTCGCCGATGAGCATGGAAGAGAGACTGGTAATCCCCCATGGAGTAATTCTCTGGATAACCACGATGGAGTGATTCCCACCTATCATTTTTCCCACGATCATGTTCTCGCTACCGCTGATGATTCAAGCACTCGAGTTGTCAAGGTTGGAGGCAGAGCAGCTGCTGTGGATCATGACAAGCTTGATTGCAAGAGAAGGTACGAATTCATAACCTTCACGAAAGTACTGTGGAAAACTGTTGTGATGCATGAACTCTTTAAGCGTCTCAGGAAGAACGACGACCGAGCGAGTGGAGGGAACAGTGTTGGAGAAGGTGCTGCAGAGCCCCACTCCGTGATGCAAGCCTCCTCCTCCGTGGGATCTGACATCTCTGGGGATGGCTATCACTGGAGAAAG

> NC_088341.1WRKY transcription factor SUSIBA2 [ Musa acuminata AAA Group (dessert banana) ]

AGTCTTCTTTTTGATGTTCAGCCCTAAACTCTGGACCAAGTAAGTACGTGACCATCTGAATTTAGAGAAGACTACATTAATTCTAGCCGACGTCATATGTCACGAAATAGTGGATCCAAGCCCAATGGTTTCATACTACAACTTGTCTTCTTTCAAACCTAACGTTCTTCATTATGGTTTCATGCATTTCAACTGGTGGTTGTTTTGGCCCTTTCGGTAGCTTTTTCTCCATCAGCCTTCCTTTCTCAGCACCACTACCAGTCTCCTCTCCGCTTAAGCATCCATTCACGAGTCCCTTTCTCTTTTCTCCTTCCTTTGTGTCTTGACACAAGTTAACTAAAGCTAGACGCTTTTATCTCTTCTCCTATCTCGACGTGTATACTCGAGCATTGCAACTTTTGTTTTGTGGTTGATGGCACGTGAATGACGCAAGTGTTCATCAGATTATGATTTCTCGCCGCTGTGGATGCCTATCTGGAGTTCGTCCTCCTCGCTCCCTGTTCTCATCTTAAAGCTGCGAGGGGGTTTTGGTTACGCTTCCTTAAGAAAAGGTCTCCGGGACTCTCTTCGATCGGTACGTAACGTGACTTTGGTATGTTGGATTCTTTTCCTCTGTTAAGACTCCTCTTTCCTTATGGCGTCTTCTTGCAACGGCTTTTATTTACTCATTTGTCTTTAATTTTGTCCCCCGCCTTCTTCTCTCTGTGCGTGTTCATGCGGCGTTGATGTGAAAAACTAGAGTTCAGAAATGTTGGAGCTTTCTATCTTCGGCCTGAGTGGAGTCTGATATATGCTTTCTGTGCAGCAGATATGTGCAATTATTAGAATCATACATTATCTGAACGTCCCAAGTTCAAGTTGCCAACTTAATCTACAATCTCATGCTTGTAGATTTGGTTTCAAGCTTGGGTTGCACGAACGATCCTAACATTCATACCTTAATACTTGAATAGCATGGTTTAGGATTCATTACGCTTGCGGGAGACATCATGAAATGGCTTTTTCAGTTTCGACCCAAAACAAGAAGATTTTACGAAACATTGATAGAAAATTTTCTTTCGAAAAATAGAAAGCATGAGAAGTTTGAGGGTAAGATATCCTGTGAATCTGCAGGAAGGAACTTGCAAACAGAAATCCCGCTTCTGGACTTGGAAGGACCAGCGTTTCTCCGATCCTCGATTAGACTGTATTAACACATTGAATTCTGAATAGCGCTTTCTCGATCTATAATTTTAGTTGCTTGTGATCTCGAACCGTCGAGGTCTCTTGCTCGGCTCCTGGCTCCTACTGGATTCCTGAAGCTAGAAGATGCTGGATATTTCCAGTTTGATGCAGGTGGGAAATGCGAGATGAGAAGAAGAATGATATCGCGAGACCTGTAGCGTCAGAACCTTTCCGAATTTTCAGCTTCTTTCCCGTGGTCGGCGTCCGCCATGAGTTCTCCTTCATCTTCTTTTGCCATCAAACCGAAGGCTATGAAATTTGATCCAACATCGAACGATCGCGCAGCTAAAGCTGTC

> NC_088341.1WRKY transcription factor SUSIBA2 [ Musa acuminata AAA Group (dessert banana) ]

TAGTTTGTCATTGCCAATATACATATAAAAATATACTAATAGTCATAGTTAGCAATGATTAACTTTAATTATTATTTTTTCTTTCGATGAATGTCAATGGAAAAAAGAAAGATCGGACAATCATTGACCATGTCCTCTCACGTTTAATTCACTACGTATTATAATATGCTCACCAATGTCTCTTTGGAAGAAGACTTAGAAGAAACCGATGAGCCCCACCACAGCCGCAATACCTCCCTTGGGTTTCTCCATCAGAATAAAATGGTACGTCCTGCCATCATTATTACCTAAGAAATCAAAGCCCACAACTTCTGATTGCTCGCCTGTGGACGAACCCCACGTCGTACTAAAATATGCTGCACTTAACCTCGGAATAGTTTGAGCTGATGTCACAACCACGAACGATTGATTAGTAGCCAACAATTCACTCGATCGACAACTTCATAGGTTCACTCGTGAAGGAGATGTATGATTTATGAACTAAATAGTAGCATGGTGAACGTATGATTGATTAGTAAACGTTAGGTGTATTTGATGCATCAAAAGGATCGAATGAGACAAACTGTGGGTTGGGTAATGTACCACCTACTCACATGAGCTCCTCGATTCAAGAATCATTCCATGCTTTTCTTAAACTCAAATTGCTCATATTTTAGACTCCATGCATTCATTCATTGTAAGGCAATTCGATCATTCATTTACTGATTTTTATTTTTAGATTTACTAATGCTTCTTTTTTTCTTTAGAAAAAAATCCTTGTTTTTATGATATTACAATAATAATAATAATAATAATAATAATAATAATAATAATAATAATAATATGCTGTCATGTTGTTATGAAGAAAAAATAATGTTACAATTTCATTGATAATGAACGATATCTATGATGATTATTATTTTAATTAGTAAAATAAATGAAAATCAAATTCAAATATTATTAGTAATCATGAAAATATATGTAAAAGATATATATTAATGTATCTTCCCACAGCTACGGCTTACTGACACCACAAACTGTACCCACAGCTTTCGTACCCACTTTTCTACCTCATTTACTCTCCCCCTCAATGATTTCTTAATCACAAGTTTTGATCACTAATCATTCGCTTCTGTGAGGGAAGACTCGGATCACGTGAATCTCTCATGCTCTTAACATGGGGAACGAACAGGGGGCATAACAGTCAATTCAACCGGTGTCTCGTTCGCCTGAATCGCGAGGGGCATCTCGATATTTCAAGTCGTAGGTAGGGGTAGTTATGTCAGTTAGAAATCCCCTTCAATTAATGGGGCCTTTCAAGTCAACCGCGTCGCAGAGCGATTGTCGCTGACAAGCACTGTATTTCCCAAATTGCCCCTCCCATCATTGCCTAAATCCCCCGACCTGTCCGCGCCCTTCGGTTCTCTCCGTCTTCCCCACTGTCACTCTCCCCTACCTCCTCACTTCCGGTTGGATCCAGAAATGGAGGGGAATCCCGAGTCGGCGGCGGGAGATCTCGGCTGCGGAGCGGCGGAGAGCCGTCCTCCGGCGCCGGAGCTGCGATTCGAGGGATCCGGCGATGGGAAGGAGGGGAGGCCGGATCCTGGGGCTTCTCCGCTCGCCGGAG

>WRKY transcription factor 21 [ Musa acuminata AAA Group (dessert banana) ]

TCTAATGGTGCAATCGAGGGACCACTAATTTTCCCTCATCATACTCAAACACTAGATAAGATTGAAACACATAAGATGTTTGGCCATTCCCAATTACCAATCAGACATGCCTTTGTTCACCATTCATTCTCAAAGTCACATGCATGTCATTGCTCAATAATTGAAGCATGAACAAGAACTAATTGCAAGGTTGGAAAAGACACCAAGGCTTTCCAAATTCGTCGACCTAACTTTGACCTTTCAAATCCCAAGATCTTTTGACGAATGCCATCATTTCATCTAAAACGATGTCAAATATAATTGGCATCTAGATTATTGTCCGACATAATCATCTCCGATGCTTGGATTCCGAGCCACCTCTCGATCATCTCGTCAAAGTTGCTTAAGGGAATCTTCTTCTTTTTCTTTCTTTCTTTCTTTTATTTTTGGTTCATCGAAAACTGCTCCATTGACTTTATGCAATGCCAAAGTACATTAGGCTTCAAACTAAGATCACTTGAGCTTTAAATTTACAGCATATATATATAATATATATAATAGGAGTATTAATGATCATATTGACAAGGATTTATGTGCTAGAAATAATTGATATGATTAAAAACAATTATGGGTGGAACTACAATTATATGAATTGATGATTTTTCAGTAAGGTGTATAGAAGAATATATGCTTATGTATAACTAAACATGATAGTAAAAATTAGAAAAAAAATAATTTAATTTTAAATTTGTGCTATATATAAAGAAAGAAAGGATGGAGTTGCAACTTGTTATTTAAGTGCTGTTGTGTTGCATTGTTGCCACAACATCAATTTTAACTAAATTATTTAATATCATAAAACAGTATATCAATAAAATACACTAATTTAAATGGGCATTAATTTTAAAATATTACTTCTAATGAATATATAATATGTGGTGAGAAGCGGGTCCATCATTTCTGGGTTCCACTTGTGCTAGGCAATCACTACACGGGTACGAATTTGGGTGTACGGATCTGGAGTATATGTTCACTACGTTTCCACTTAATACGTGGGTGGTGAGAAATTAAATAATATATAAATAAAGGGACTGAAACGCATAAAAATACTCATGCTCTAAATTATAATGCGCCTCCTATATTTCCAAGTTTTAACACTTTAGTCCTCGCGGTCGGGTATTAAAGTACTAAAGTGGAGGAGGAGGCGGAGGAAAATTCCAACGCGGCTCATGTTGTTTCCCTCACGCCTTCCAATCCCACGGACGCCATAGGAAGACCAACACTCTAGGGTTCTTCCCCGCCACTTTCCCAAGTCCAAGGTCAGCTCTCTCATGCCCCCCTCTTATCCGCCCGTTTCCTTGCCGTGGTGGATTTGATTCACTCGGCTAG

> NC_088335.1WRKY transcription factor WRKY51 [ Musa acuminata AAA Group (dessert banana) ]

TCGTATCGTACCGATATTTCGATGTTGACTCGGTACGGTATAATACCGACGTATCAAATGGTACATCAGGACATACCGAATAATTTTATATATATATATTTTTTTTTACTGTAATACTGTAACACTGCTACTATAGTACTGTAGCACTATAATACTATTGCACGTTCCATCCGATATTGAGCGATCCACGTACCGATATATTATCAAACTGATACGCATCGTCAATATCAAACGGTACCATTTTGTACTGCATAGCATGATAATTTCATTTGTGCATGATAAGATGTTCAGACCATGATGACATTTTAAGTGAATTATTGCTTATAAGGTGATTGCATGTTCAATGCATTGCTTTTGGTTAGATCTCAATCAAGGTTAAGTCAAGCCTTACTTATATAGAAATCTTACCAAAATCTTTTTTTTTTCCTCCAAGCAATCCATTTGAAATAGCCAATTTTAATAGAACCCAAGTGAGAAGATGAAGAATTTGATTCCCAATAAAGTAGGAAGATTTCATGGCCTAATTGTATTTTATTACTTTGTACTTGCTGACCATACAAATAATTGAATCTTTTAACAGGGTAGTCCATGGGCTTGTTTGAGCATATCAGCCTATTTAAGGCTCATCTGCATAGCAATAAATGAGAGCAAAGGACATATTATTTAATGGCCATACAAATAGTAGAGTGAGACAAGCAACCACAATATCCTAAAATAACTCCCTCCCTTGGAATTTCCAAATTGCATATTTGAATTAATAAAATAGAATTATGTTAAAAAAAAAGCTATTTCAAATATGAAATAAAGTCTTTTAGAATTCTAGTGTAATTTTCAAAGTATACAATATATCGTACCGATATTTCGATGTTGACTCGGTACGGTATAGTACCGACGTACCAAATGGTACATCAGGACATACCGAATAATTTTATATATTTTTATTTTACTGTAATACCGTAACACTGCTACTATAGTACTATAACACTATTATAGTATAACAATGTAACATTGCTATAGTGTAGCACTATAATACTATTGCATGTTTCATCCGATATTGAGCGATCCGCGTACCGATATATTATCAAACTGATACGCATCGTCGATATCAAACGGTACCATTTGGTACTGCGTAGCATGATAATTTCATTTGTGCATGATAAGATGTTCAGACCATGATGACATTTTAAGTGAATTATTGCTTATAAGGTGATTGCATGTTCAATGCATTGCTTTTGGTTAGATCTCAATCAAGGTTAAGTCAAGCCATTCAATATTAGCTATTCAAATCCCATGTGTACAATTTCTTTTATTTAACATAAATAAATTGAATACATCATCTGGATTACAATCTGCATGATTTTTTTATTTTTTAAATATTTTGTAGGTTTATTATAATTTATTGAGAAGTTGAATAAGAGCATACGGATCAGCAAAAAGGAAGTCAGACAAAGTCTACTCATCGAACGGCTGATAGCGTTTTGAAACCTCGTGATTGGTGAAAGGACTGACGTCGATGTACCACTGCGGCTTTTATTACTTGCAATTGCTTTTCTTACTTAAAAAACACACACGCGATGTCTACCTGCCTTTTTATTGGAGAACGAGAGCTCTGCAGGCAAGCCCCAACTACTACCCAATCACCTAACAGGTAGAAGAGGGGACGACAATGAAAGAATATATTGCCTATTTTAATTTATTCTTTTTCTTTTGTATTTTCTTCGTTTTTTAAACACGAAAGACAAGAATCCACCGTCGCACGTACCTCTGTCTAAAGGTGGGTCCCCCATCAGGTCTACCGTTTCTATTTAACAGCAACTGAGGTAGGAGATTTGTCGGGGTTGACGAGAGTGTTGCTGCGAGTTTCGGTCTCAACGGAAG

> NC_088334.1GRAS family protein RAD1-like [ Musa acuminata AAA Group (dessert banana) ]

CTTTCTTATATCATTTTCTGAAGTTATTTTCTTCATAAATTGTATCGATACACATCCAGCATCCACATTTCTATAATGATCGAGCCAGTTATGTATGTTAGGGTACGAGCATCACTCACCGGAAAACTTAGCCATGCAAACATCGTAGAGCAAATAAGTAGTCTGCAGGCTTTGTTGAGAGATTACGTTCTCCTCGAACAAGAAACCTCTCGAGTCTGTAATAACGAATGATGGGCGAGCAGATTCCTTCGAGCTATGCCGACACACTCTTTGTTTATGATTTCGACTCTTTGGAAGATTTCAATCGCAAGATTCTCCCTCAAACAATGTACACGAAATGGTGAGGATACAACACGCACACAACATTGTTCCTAACGAAAGTGTAGGATATCCGGATTCCTCCACCATCGACTCCTTGAAGCAACCAAGTATTCCTGGAATAATCTTTTCCTGTTTCGCTACGATTAATCATGCTTACACATCAAGCATGATTAATAAACCACTTCCTTGTGTCAAAAACGTCAATACCACATCACTTCCTGTTGGATTCTGACCATGTCAAGAAGGGTGTGACCAAGAATCCTAATTGTTTTGTCATGGTTTCATTAGATTCTTTATGGTTGATGGCCGGCTTCTTGTCTCCCTTCAGAACTCGAGGAAGCTTTTTTATTGCGAGAGATGAGCAGTGTTCCCTGATGGTGAGAAAACGAGTGGTGAAGCATATTGTACTCCCCATTACTGCTGGAGTTAATGCTGCAGCGAAAGGGAGCACGACTATTAAGATTGCTGGTTCTTCTCACCGTACGGGATTCGTGTTGCAGTAGCTTACGCCATTGACGTTGTCGCCCAATTATGTTCTTTTGACTCATGTCGATAGATCTTTCGATACGTAGATGCATAAAGTGACCGACGTTTTGCTGTTGTCTTGACGTCGAAGTTTGTCAATCGAGGAGCAGAAGTAAAAGCTTTACTTGTGCTACGATTTGTTAGCTCAGAGAGAGAGAGAGAGAGAGAGATTTATAAGCTTTACTTGTGTTGCGATTTGTTAGATCTGAGAGAGAGAGAGAGAGAGAGATTTATAAGCTTTACTTGTGTTGCGATTTGTTAGATCTGAGAGAGAGAGAGAGAGAGAGAGAGAGAGAGAGAGAGAGAGAGATATTTAAAAGCTTTACTTGTGTTGCGATTTGTTAGATCTGAGAGAGAGAGAGAGAGAGAGAGAGAGAAAGAGAGAGAGAGAGAGAGATATTTATAAGCTTTACTTGTGTTGCGATTTGTTCGATCTGAGAGAGAGAGAGAGAGAGAGAGAGAGAGTTAAGCTGCGATATCCAATGTCTCCTGCATGCTACTTGAAGCCTCAAGAAGAACAACTGCAACAGTTGAATTACAATGAGGAGGGGATGGGTGACAATGGCGCCGAGCTCGGCCTATCGAACATGGCTAAAATGGATCTGCATCCAGTCTGCTTTAACC

> NC_088332.1GRAS family protein RAM1-like [ Musa acuminata AAA Group (dessert banana) ]

ACGCTAACTGTCTGTACAATAAGTGAGGATGACGAGGGTGAGGTGTTGAGTCTAATGAAAGCCGAGGAAGAAGATAGAGATAGTAACAGGTTCAACGGGGCAAAGGAGAGGGGAGGTTATGATGGTACAAGGCGGAGATAGAGACAAATTCGACAAGGGCCAAAGATGGAGGGGGTGGTAGAGGCAAATTCGACGGGGGCTAAAGGGGGAGGGGATGATAGGTCTAGTAGGATAGAGAGGAGGGGAGGTTATGAAGACACAGGATGGAGGTGAAGATATATTCAACAAGAGTTAAGAGTAAAATGTTCATTTATAAGAAAAGATATTTTATGAAAATTTTTTTAAGTTGGAATATGGAAATTTTTTAGAATAATAACTTTTTTTATTAATTCGCCCGTCTATGCAATCACTTTATAGTCTGACATAGGATTTAAGGACAATTTCTCCTGTCAGCAGGACTAGTAGGGACTTACTACTGTGTTATAGCTGTGTAAGTGCTCGTCATTTTTCCTACACATTTGTTCTTTTCTCTGGGCAATACTGTTGCTGTGGAAAGACATGGAGTTATATTACACACTTAGAGATGAATCATTGATGAGTGTAGACAGCTTTGGTGTCCAACAATTGACAGGTTCAATAACAGGCTAAACAATGTTCAAGCAATTTGGCTGGTAGTCCAAGCTGTCCCAGCTCAATCGATAGCCTCCTTTCACTTCTATTGTATACCTTGACAAGGTCTTTAATTTCCCTCAAAGAAAAGGAATCACATTGTTTGTAGTAAACAAGTGGGTAATACATGCATGGCTTGTTTTGGCTCGATCGTAGGATACAATTCCAGCTAAAGAATGAGCTACAAAACAAGAGAAGCGGATAGAACCGATGAGAAAGCCCGCACGGCACGAATCCAAGCGCTCCAATCCTGTGGCAAATCCAAGGACCAGGCGGCGCCATCGACGCCACTACCGACGCCACCGTATCACCTCTCTCTCCTTCCTCTGTCACTCTCACCAGCTTCTTAGGGCATCACACAACACTTGTCTTCGTTGCTGCCACAGTTGTCTATCCCAAACTGGTTAGTTCCTTGTGCCGGCCCTATGCATTGGGCGTTAGGATGCAAGCTCTCATTCAGCAAACAAACAAACTATCGAAGAGGAACAGAGCTCCTTTTTAAACAAGCAAAATGGTTCCTTTGAACTCTTTTGATTGGTAGGAATCGGGAGTCCAAATGCCTCCAAACGCTTTAGGTTAGCAGCCGGGTGTTACCATCAAATTCCCTTCCTGCAATGATACACGGATATCCTTGATCGATATCCTTAAATCACCCTCCTTACCTGCCTGCTATTAGTCATCAGCTAAGGTGGAGTTCCCATGAGAAGAACCCCACGCTTAGTCTGAGATCA

> NC_088356.1GRAS family protein RAD1-like [ Musa acuminata AAA Group (dessert banana)

GGGGGTGGCTTTTATACCTGCGAACGAGGATCGGTCATACATGGTCCGTGAATGGTTGCCGACTCCTCGGGCAATTGGCTTGTATCTTACGTGTAAGCATCGACTAACCTGTATAGACAAGTGTCGTGTAGTATCGTTTCGAGCTTTCTAGAATGGCTCTGCGGTATGCAGCGCCGACTTGTTCGGTGTCGAATAGTACGAGGGCAGGTGATTGTCCTGGAGATCTTTTATTAGCACTGTCATGACCGTCGACCGCTGCCACATGGAGTGCTGAAATATGCCATATCACATTGAGGTTTGAAAATAACTTCACATAAAATTTCAGATATGGGTTGTGTCTCGTGATCCAATTCATATCTCTTCCTGGATTCACCAACGAAGCTGATGGTTCCTATCAGCGGTTGTTCCTCAGAAGCAGGAACGACCTCCTCTTTTGTAGATGAGGTTTAATCGGAAGGTTGAAGTAGTTGATCTGCAATCTCTTTTAGGACGTTCGATTCTTTCTCATATCATTTTCTGAAGTTATTTTCTTCGTAAATTGTATGGATATATATCCAGCATCCACATTTCTATAATGATCGAGCCAGTTATGTATGTTAGGGTACGAGCATCACTCACCGGAAAACTTAGTAGTCTGCAGGCTTTGTTGAGTGATTACGTTCTCCTCGAACAAGAAACCTCTCGAGTCTGTAATAACGAATGATGGGCGAGCAGATTCCTCCGAGCTATGCCGACACACTCTTTGTTTATGATTTCGACTCTTTGGAAGATTTCAATAGCAAGATTCTCCCTCAAACAATGTACACGAAATGGTGAGGATACAACACGCACACAACATTGTTCCTAACGAAAGTGTAGTATATCCGGATTCCTCCACCATAGACTCCTTGAAGCAACCAAGTATTCCTGGAATAATCTTTCCCTGTTTCGCTACGATTAATCATGCTTACGCATCAAGCATGATTAATAAACCACTTCCTTGTGTCAAAAAAGTCAATACCACATCACTTCCTGTTGGATTCTGACCATGTCAAGAAGGGTGTGACCAAGAATCCTAATTGTTTTGTCATGGTTTCATTAGATTCTTTATGGTTGATGGCCGGCTTCTTGTCTCCCTTCAGAACTCGAGGAAGCTTTTTTATTGCGAGAGATGAGCAGTGTTCCCTGATGGTGAGAAAACGAGTGGTGAAGCATATTGTACTCCCCATTACTGCTGGAGTTATGCTGCAGCGAAAGGGAGCACGACTATTAAGATTGCTGGTTCTTCTCACCGTACGGGATTCGTGTTGCAGTAGCTTATGCCATTGACGTTGTCGCCCAATTATGTTCTTTTGACTCATGTCGATACATCTTTCGATACGTAGATGCACAAAGTGACCGACGTTTTGCTGTTGTCTTGACGTCGAAGTTTGTCAATCGAGGAGCAGAAGTAAAAGCTTTACTTGTGCTACGATTTGTTAGCTCAGAGAGAGAGAGAGAGAGAGAGAGAGAAAGAGAGAGAGAGAGAGATATTTATAAGCTTTACTTGTGTTGCGATTTGTTAGATCTGAGAGAGAGAGAGAGAGAGAGAGAGAGAGAGAGAGAGAAAGAGAGAGAGAGAGAGAGATATTTATAAGCTTTACTTGTGTTGCGATTTGTTAGATCTGAGAGAGAGAGAGAGAGAGAGAGAGAGAGAGAGAGAGAGAGAGAGAGAGATTTATAAGCTTTACTTGTGTTGCGATTTGTT

> NC_088354.1GRAS family protein RAM1-like [ Musa acuminata AAA Group (dessert banana) ]

CTTGGCACAATACCCTCTCTTCTTGGCCCGATGCCCGAGTCCACGGCCCGAAGCCTTCTGTCGATACGTCGACCGATCCATCGGCCCGACGTCCAATCTTCTGACATGTTCCTTCGGCACAACATGATTTTTCCTGCTTTAATTGTCTCATCCTGATCGAAGCATCCTGCATCACTCAAAACACAGAATAAATCATAAACATATATCAAGTGGTTTCATCATCAAAATACGAGATTCAATAACCTCCCCTTTTTGATGATGACAACCACTTGATAACGGAGTTAACCTTAACTATCGGAGTTTAAACAAACTCCCCCTATCAATATGCCATATTGATAGAAACTCTTGGATTCAAAAATTCAAGCAACATGTCATCATATACTTATGCATAACATGTCATGTTATCAACATACTTCTCCCCCTTTGTCATCAACAAAAAGGAGATGTACAACTATTCTTGTGTTTGTGATATAAGTTCAACTCATTGCATGAAAAACATAATATCAAGTTTTATCATCATGTAGTTTTGAAGCTAGAAAATTTAGCAAGTGTTACATCATGCTTAGAACATTCAAGCTATCAAGTTTTAGATATGCAAGTTTTACAACATACAAGATAGCACTTTTGCTTCTTTTGAGATAGCAACTTCTACATCATGCAAGCTAGCAAATTAGAGATGTTCAAGAAGGCAACTCTTGCTTCTTGAAATATGTAAATTTTGTCAACTTTGCTAGATGCACAAGTTAGCATGTTTTGCTTCTTGAGATAGGCAAGATAGCACTTTTGTAATTTTTGTTTCTTAAGATGTTCAAGATAGCAAGCTCTACATCTTGCAAGCTAGCAATGTTACAACATTTTAGAACATGCAAGCTAGCAATTTAGAGATGTTCAAGAAAGCAACTCTTGCTTCTTGAAATATGCAAGTTTGCTAGATGTGCACGTTAGCATGTTTTGCTTCTTCTTGAGATTTCAAACTAGCAATTATCGGTGACGTTCAAGATAGCAATTTCTTCTCTTTTGAGAAGTGCAATTTTTGCTTTCGTCTTGAATTGTGCAAGCTAGCTAGTTTGTCTCTTTTCATGATATTCATGCTAGAAATTCTTGCTCCCCCTTTGTCATTGTCAAAAAGAAGGGAAGACCCTTATATCAATTTTCAGATTATGAAAAAGGTAAGTATCAATCTTATTTGTGCATCATTATATATTCAAATTAAAATATACACAATTTCAAAAACCTTATTTTTCATGCACGATACCAAAAAAAAAATCAATCATCATTAATGGGCATATCATACATGATACTCAAACATTAAAATTTTTAGCATTTATCAACATGTTGATCATGATACCAAACATTCATTATTTAAAGTATTTATGATACACATCATATGCAATACATCATGTACATCATTGTCATAAGTATTGCATGCATCATTTCAAATTCATGAATATTTCATCATTGCATGCATCATACCAAATCATAAAATATAAAATCATTATTAATACATGATTCCAAATTATCATTTATTTCACATCATAATGGTACCAATTTTGTCAAATTACATCCAACCATGCATGATCGAAACTTCTTATCAAAACAAATTAATGAATATTTCATGTATTCATATCATCACATATGGAATATCAAGAAGAATTATTAGGTGATGAGATGTATTTCATTCATTCAAAGAATTTTATATAACAAAATTCAAGTCATAAGCCTTAAAAGATGTCATGAGAGAACATATAAACAATCTTGATTTATAAAATGAATACTCAAGTTATAATTTCGAGAAATCAAGAGAAATCGTGATTCATTTTAACAAATCTCCTCTTAAATAAATAACATAAAGAATTCTTAGGAGATCATGAAAAAGAT

> NC_088345.1GRAS family protein RAD1-like [ Musa acuminata AAA Group (dessert banana) ]

GCGAACGAGGATCGGTCATACATGGTCCGTGAATGGTTGCCGACTCCTCGGGCAATTGGCTTGTATCTTACGTGTAAGCATCGACTAACCTGTATAGACAAGTGTCGTGTAGTATCGTTTCGAGCTTTCTAGAATGGCTCTGCGGTATGCAGCGCCGACTTGTTCGGTGTCGAATAGTACGAGGGCAGGTGATTGTCCTGGAGATCTTTTATTAGCACTGTCATGACCGTCGACCGCTGCCACATGGAGTGCTGAAATATGCCATATCACATTGAGGTTTGAAAATAACTTCACATAAAATTTCAGATATGGGTTGTGTCTCGTGATCCAATTCATATCTCTTCCTGGATTCACCAACGAAGCTGATGGTTCCTATCAGCGGTTGTTCCTCAGAAGCAGGAACGACCTCCTCTTTTGTAGATGAGGTTTAATCGGAAGGTTGAAGTAGTTGATCTGCAATCTCTTTTAGGACGTTCGATTCTTTCTCATATCATTTTCTGAAGTTATTTTCTTCGTAAATTGTATGGATATATATCCAGCATCCACATTTCTATAATGATCGAGCCAGTTATGTATGTTAGGGTACGAGCATCACTCACCGGGAAACTTAGTAGTCTGCAGGCTTTGTTGAGTGATTACGTTCTCCTCGAACAAGAAACCTCTCGAGTCTGTAATAACGAATGATGGGCGAGCAGATTCCTCCGAGCTATGCCGACACACTCTTTGTTTATGATTTCGACTCTTTGGAAGATTTCAATAGCAAGATTCTCCCTCAAACAATGTACACGAAATGGTGAGGATACAACACGCACACAACATTGTTCCTAACGAAAGTGTAGTATATCCGGATTCCTCCACCATAGACTCCTTGAAGCAACCAAGTATTCCTGGAATAATCTTTCCCTGTTTCGCTACGATTAATCATGCTTACGCATCAAGCATGATTAATAAACCACTTCCTTGTGTCAAAAAAGTCAATACCACATCACTTCCTGTTGGATTCTGACCATGTCAAGAAGGGTGTGACCAAGAATCCTAATTGTTTTGTCATGGTTTCATTAGATTCTTTATGGTTGATGGCCGGCTTCTTGTCTCCCTTCAGAACTCGAGGAAGCTTTTTTATTGCGAGAGATGAGCAGTGTTCCCTGATGGTGAGAAAACGAGTGGTGAAGCATATTGTACTCCCCATTACTGCTGGAGTTATGCTGCAGCGAAAGGGAGCACGACTATTAAGATTGCTGGTTCTTCTCACCGTACGGGATTCGTGTTGCAGTAGCTTATGCCATTGACGTTGTCGCCCAATTATGTTCTTTTGACTCATGTCGATACATCTTTCGATACGTAGATGCACAAAGTGACCGACGTTTTGCTGTTGTCTTGACGTCGAAGTTTGTCAATCGAGGAGCAGAAGTAAAAGCTTTACTTGTGCTACGATTTGTTAGCTCAGAGAGAGAGAGAGAGAGAGAGAGAGAGAGAGAGAGAAAGAGAGAGAGAGAGAGATATTTATAAGCTTTACTTGTGTTGCGATTTGTTAGATCTGAGAGAGAGAGAGAGAGAGAGAGAGAGAGAGAGAAAGAGAGAGAGAGAGAGAGAGATATTTATAAGCTTTACTTGTGTTGCGATTTGTTAGATCTGAGAGAGAGAGAGAGAGAGAGAGAAAGAGAGACAGAGAGAGAGAGAGAGAGAGAGAGAGAGATTTATAAGCTTTACTTGTGTTGCGATTTGTTAGATCTGAGAGAGAGAGAGAGAGAGAGAGAGTTAAGCTGCGATATCCAATGTCTCCTGCATGCTACTTG

**>**NC_088343.1 GRAS family protein RAM1-like [ Musa acuminata AAA Group (dessert banana)

ATTCGACAAGGGCCAAAGATGGAGAGGGTGGTAGAGGCAAATTCGACGGGGGCTAAAGGCGGAGGGGATGATAGGTCTAGTAGGATAGAGAGGAGGGGAGGTTATGAAGACGCAGGATGGAGGTGAAGATATATTCAACAAGAGCTAAGAGTAAAATGTTCATTTATAAGAAAAGATATTTTATGAAAATTTTTTTAAGTTGGAATATGGAAATTTTTTAGAATAATAACTTTTTTTATTAATTCGCCCGTCTATGCAATCACTTTATAGTCTGCCATAGGATTTAAGGACAATTTCTCCTGTCAGCAGGACTAGTAGGGACTTACTACTGTGTAATAGCTGTGTAAGTGCTCGTCATTTTTCCTACACATTTGTTCTTTTCTCTGGGCAATACTGTTGCTGTGGAAAGACATGGAGTTATATTACACACTTAGAGATGAATCATTGATGAGTGTAGACAGCTTTGGTGTCCAACAATTAACAGGTTCAATAACAGGCTAAACAATGTTCAAGCAATTTGGCTGGTAGTCCAAGCTGTCCCAGCTCAATCGATAGCCTCCTTTCACTTCTATTGTATACCTTGACAAGGTCTTTAATTTCCCTCAAAGAAAAGGAATCACATTGTTTGTAGTAAACAAGTGGGTAATACATGCATGGCTTGTTTTGGCTCGATCGTAGGATACAATTCCAGCTAAAGAATGAGCTACAAAACAAGAGAAGCGGATAGAACCGATGAGAAAGCCCGCACGGCACGAATCCAAGCGCTCCAATCCTGTGGCAAATCCAAGGACCAGGCGGCGCCATCGACGCCACAACCGACGCCACCGTATCTCCTCTCTCTCCTTCCTCTGTCACTCTCACCAACTTCTTACGGCATCACACAACACTTGTCTTCGTTGCTGCCACAGTTGTCTATCCCAAACTGGTTAGTTCCTTGTGCCGGCCCTATGCATTGGGCGTTAGGATGCAAGCTCTCATTCAGCAAACAAACAAGCTATCGAAGAGGAACATAGCTCCTTTTTAAACAAGCAAAATGGCTTCTTTGAACTCTTTTGATTGGTAGGAATCGGGAGTCCAAATGCCTCCAAACGCTTTAGGTTAGCAGCCGGGTGTTACCGTCAAATTCCCTTCCTGCAATGATACACGGATATCCTTGATCGATATCCTTAAATCACCCTCCTTACCTGCCTGCTATTAGTCATCAGCTAAGGTGGAGTTCCCATGAGAAGAACCCCACGCTTAGTCTGAGATCAGTTCCCTTGTTGAGGTT
